# DDX3 DEAD-box RNA helicase plays a central role in mitochondrial protein quality control in *Leishmania*

**DOI:** 10.1038/cddis.2016.315

**Published:** 2016-10-13

**Authors:** Prasad Kottayil Padmanabhan, Ouafa Zghidi-Abouzid, Mukesh Samant, Carole Dumas, Bruno Guedes Aguiar, Jerome Estaquier, Barbara Papadopoulou

**Affiliations:** 1 Research Center in Infectious Diseases, CHU de Quebec Research Center-University Laval and Department of Microbiology, Infectious Disease and Immunology, Faculty of Medicine, University Laval, Quebec, QC, Canada; 2 Department of Zoology, Kumaun University, Almora, Uttarakhand, India; 3 CNRS FR3636, Université Paris Descartes, Paris, France

## Abstract

DDX3 is a highly conserved member of ATP-dependent DEAD-box RNA helicases with multiple functions in RNA metabolism and cellular signaling. Here, we describe a novel function for DDX3 in regulating the mitochondrial stress response in the parasitic protozoan *Leishmania.* We show that genetic inactivation of DDX3 leads to the accumulation of mitochondrial reactive oxygen species (ROS) associated with a defect in hydrogen peroxide detoxification. Upon stress, ROS production is greatly enhanced, causing mitochondrial membrane potential loss, mitochondrial fragmentation, and cell death. Importantly, this phenotype is exacerbated upon oxidative stress in parasites forced to use the mitochondrial oxidative respiratory machinery. Furthermore, we show that in the absence of DDX3, levels of major components of the unfolded protein response as well as of polyubiquitinated proteins increase in the parasite, particularly in the mitochondrion, as an indicator of mitochondrial protein damage. Consistent with these findings, immunoprecipitation and mass-spectrometry studies revealed potential interactions of DDX3 with key components of the cellular stress response, particularly the antioxidant response, the unfolded protein response, and the AAA-ATPase p97/VCP/Cdc48, which is essential in mitochondrial protein quality control by driving proteosomal degradation of polyubiquitinated proteins. Complementation studies using DDX3 deletion mutants lacking conserved motifs within the helicase core support that binding of DDX3 to ATP is essential for DDX3's function in mitochondrial proteostasis. As a result of the inability of DDX3-depleted *Leishmania* to recover from ROS damage and to survive various stresses in the host macrophage, parasite intracellular development was impaired. Collectively, these observations support a central role for the *Leishmania* DDX3 homolog in preventing ROS-mediated damage and in maintaining mitochondrial protein quality control.

DEAD-box proteins form the largest family of RNA helicases and are conserved from bacteria to humans. They belong to superfamily 2 (SF2) of RNA helicases which harbor an Asp-Glu-Ala-Asp (DEAD) motif that defines the family.^1^ DEAD-box RNA helicases are central players in RNA biology and function in essentially all aspects of RNA metabolism. With few exceptions, little is known about how these enzymes physically perform multiple cellular tasks.^1^ *Leishmania* and other Trypanosomatidae encode 48-50 DEAD-box RNA helicases^2^ as opposed to 25 in yeast and 37 in humans.^1^ Similar to other eukaryotes, many biological functions have been attributed to trypanosomatid RNA helicases, including RNA degradation,^3^ translation regulation,^4^ and RNA editing.^5^ We recently characterized a DEAD-box RNA helicase of 67 kDa (HEL67) in *Leishmania* and demonstrated that it prevents ribosomal RNA degradation through an antisense rRNA-mediated pathway and translational arrest triggered by apoptotic stimuli.^6^

Unlike higher eukaryotes, the protozoan *Leishmania* has a single mitochondrion^7^ that not only serves as the major site of ATP production through oxidative phosphorylation but also plays important roles in maintaining cell survival, apoptosis, and metabolic homeostasis.^8^ Thus, mitochondrion is a central sensor of stress-induced cell death in several *Leishmania* species.^9, 10, 11, 12^ It has been shown that reactive oxygen species (ROS) represent a host cell defense in inducing the parasite death.^13, 14, 15^ Excessive levels of mitochondria-derived ROS promote mitochondrial dysfunction, resulting in loss of many cellular functions and in multicellular organisms, the onset of disease.^16^ Consequently, there are several quality control systems that monitor mitochondrial protein degradation to maintain mitochondrial homeostasis, including mitophagy, protease-mediated turnover and the ubiquitin–proteasome system (UPS).^17, 18, 19^ Recently, it has been reported that mitochondrial stress in *Caenorhabditis elegans* affects UPS.^20^ However, the role of UPS is largely unknown in protozoa.

In this study, we have investigated the impact of the DEAD-box RNA helicase HEL67, the DDX3 homolog in *Leishmania,* in regulating cell death under conditions of cellular stress impacting on mitochondrial function. We show that *Leishmania* genetically depleted for DDX3 is highly susceptible to various stress stimuli encountered in the mammalian host and is thus unable to undergo intracellular development. Furthermore, we demonstrate that inactivation of DDX3 increases mitochondrial ROS production concomitantly with the levels of polyubiquitinated proteins, leading to mitochondrial membrane potential collapse, mitochondrial fragmentation and cell death. Consistent to these findings, we report potential interactions of DDX3 with key components of the stress cellular response, in particular p97/VCP/Cdc48, which is essential in clearing oxidatively damaged mitochondrial proteins.^21, 22, 23^ This is the first demonstration, to our knowledge, of a central role for a DEAD-box RNA helicase of the DDX3 subfamily in mitochondrial proteostasis.

## Results

### DDX3 is crucial to *Leishmania* intracellular growth and differentiation triggered by stress sensors

Phylogenetic analysis of the alignment of the *Leishmania* RNA helicase HEL67^6^ with members of the helicase SF2 superfamily (Supplementary Figure S1a) revealed that HEL67 is part of the Ded1/DDX3 cluster, which is closely related to DDX4, DDX5, and DDX46 and also includes the *Saccharomyces cerevisiae* Ded1 and Dbp1, the *Homo sapiens* DDX3X and DDX3Y, the *C. elegans* VBH-1, the *Drosophila melanogaster* Belle, and the *Xenopus laevis* An3 proteins^24^ (Supplementary Figure S1b). HEL67 harbors all 12 signature motifs found in the highly conserved helicase core of Ded1/DDX3 subfamily members that are involved in ATP binding and hydrolysis, RNA binding, and the link of ATPase and helicase activities^1, 24, 25^ (Supplementary Figure S1a) and hereinafter we will refer to as DDX3.

**Figure 1:**
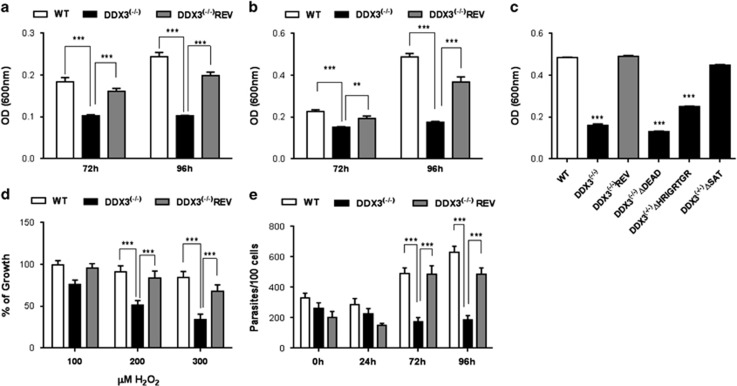
DDX3 is crucial to *Leishmania* intracellular growth and differentiation triggered by stress sensors. *L. infantum* promastigotes wild type (WT), DDX3^(−/−)^ knockout and the add-back mutant DDX3^(−/−)^REV were grown under temperature stress (37 °C) (**a**) or acidic pH (5.5) at 25 °C (**b**) or a combined heat and acidic pH stress triggering differentiation into amastigote forms (**c**). The DDX3^(−/−)^ knockout was complemented with DDX3-HA (DDX3^(−/−)^REV) or DDX3 deletion mutant proteins lacking either the LDEADRM motif (DDX3ΔDEAD) or the HRIGRTGR motif (DDX3ΔHRIGRTGR) participating in ATP binding and hydrolysis and/or the SAT motif (DDX3ΔSAT) involved in RNA binding (Supplementary Figure S1a). The parasite density was measured at 600 nm at 24 h intervals. (**d**) Parasites exposed to increasing concentrations of H_2_O_2_ (100–300 *μ*M) for up to 72 h. (**e**) Intracellular survival of WT, DDX3^(−/−)^ and DDX3^(−/−)^REV strains was evaluated following infection of mouse-derived bone marrow-derived macrophages. The results shown here are the mean and standard deviation of 3–5 independent experiments performed in triplicates. Statistical significance was assessed by two-tailed paired Student's *t*-test. Asterisks indicate significant differences between WT, DDX3^(−/−)^ and DDX3^(−/−)^REV strains (^∗∗^*P*⩽0.01, ^∗∗∗^*P*⩽0.001)

Our prior studies have established an important role for the *Leishmania* DDX3 in protecting parasites from stress-inducing cell death by preventing translational arrest.^6^ As an intracellular pathogen, *Leishmania* has to overcome various stresses in order to survive in the phagolysosome of mammalian macrophages. First, we investigated whether DDX3 impacts the parasite response to stress signals triggering promastigote to amastigote differentiation inside macrophages such as higher temperature (37 °C) and low pH (~5.5).^26^ *Leishmania infantum* genetically depleted for DDX3 (DDX3^(−/−)^)^6^ were sensitive to heat stress (Figure 1a) and acidic pH (Figure 1b) compared with the wild type (WT) and add-back mutant (DDX3^(−/−)^REV). Accordingly, DDX3^(−/−)^ parasites were unable to undergo amastigote differentiation upon exposure to a combined heat and acidic stress (Figure 1c). Complementation of DDX3^(−/−)^ parasites with DDX3 mutant proteins lacking either the LDEADRM motif (motif II) (DDX3ΔDEAD) or the HRIGRTGR motif (motif VI) (DDX3ΔHRIGRTGR) (Supplementary Figure 1Sa) participating in ATP binding and hydrolysis^24^ failed to restore amastigote differentiation (Figure 1c). On the other hand, the SAT motif (motif III; Supplementary Figure 1Sa) facilitating ssRNA binding^24, 27^ was dispensable (Figure 1c). These data suggest that DDX3's critical role in amastigote differentiation requires the ATPase and not the RNA helicase activity. Because *Leishmania* has also to cope with oxidative burst in macrophages and is susceptible to exogenous ROS,^28^ we assessed the effect of hydrogen peroxide (H_2_O_2_) on *Leishmania* growth and showed that parasites lacking DDX3 were more sensitive to oxidative stress than control strains (Figure 1d). Thus, these results explain the failure of DDX3^(−/−)^ *Leishmania* to replicate within bone marrow-derived murine macrophages (72–96 h post-infection) compared with the controls (Figure 1e).

Overall, these results indicate that growth of DDX3-depleted parasites is drastically impaired in the presence of stress signals encountered in the mammalian host.

### Inactivation of DDX3 leads to mitochondrial ROS accumulation associated with a defect in hydrogen peroxide detoxification

**Figure 2:**
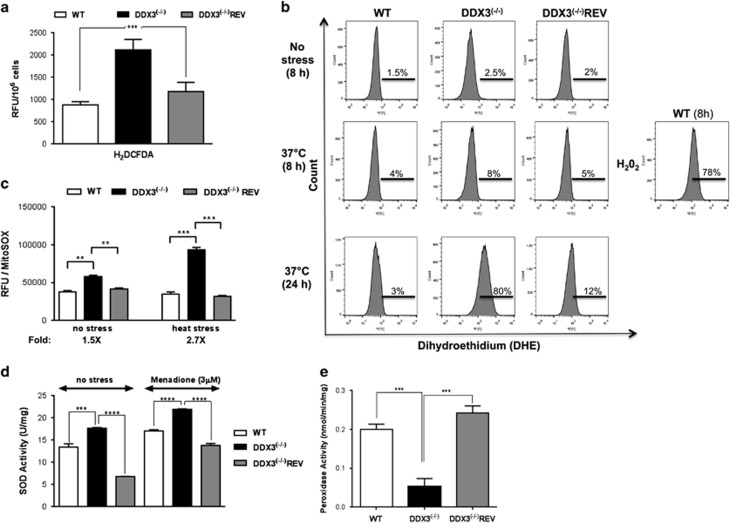
Inactivation of DDX3 leads to an increased production of mitochondrial ROS associated with a defect in hydrogen peroxide detoxification. (**a**) Production of peroxyl radicals and peroxides in WT, DDX3^(−/−)^ and DDX3^(−/−)^REV strains was measured using the 2′,7′-dichlorodihydrofluorescein diacetate (H_2_DCFDA) probe. Results shown here are expressed in relative fluorescence units. (**b**) Superoxide production in the above strains cultured at RT (no stress) or at 37 °C for 8 and 24 h. Parasites were stained with the dihydroethidium (DHE) probe and analyzed by flow cytometry. WT parasites exposed to H_2_O_2_ for 8 h were used as a control. The experiment was repeated three times in duplicate with similar results. (**c**) Mitochondrial superoxide accumulation was measured using the MitoSOX Red probe. Parasites (2–4 × 10^7^) were treated with 5 *μ*M MitoSOX for 2 h at 25 °C and analyzed with a Victor fluorometer. The fluorescence was measured at 510 nm excitation and 580 emission wavelengths. Fluorescence was normalized with protein concentration measured using Bio-Rad protein assay. (**d**) SOD activity and (**e**) peroxidase activity were measured as indicated in the Materials and Methods. The results shown here are the mean and standard deviation of three independent experiments performed in triplicates. Statistical analysis was performed using the two-way ANOVA. Asterisks indicate significance, ^∗∗^*P*⩽0.01, ^∗∗∗^*P*⩽0.001, and **** *P*<0.0001

The high sensitivity of DDX3^(−/−)^ *Leishmania* to heat and oxidative stresses prompted us to evaluate ROS production. First, we measured general cellular ROS using the 2′,7′-dichlorodihydrofluorescein diacetate (H_2_DCFDA) dye that once oxidized, mostly by peroxyl radicals and peroxides, emits fluorescence directly proportional to the amount of ROS. DDX3^(−/−)^ parasites produced 2.3-fold higher levels of oxidative species compared with the controls (Figure 2a). Superoxide production was measured by flow cytometry using the dihydroethidium (DHE) dye. Unstressed DDX3^(−/−)^ parasites demonstrated a slight increase in superoxide levels but upon heat stress (24 h), 80% of DDX3^(−/−)^ accumulated superoxide compared with 3 and 12% for the WT and DDX3^(−/−)^REV, respectively (Figure 2b). Oxidant levels in DDX3^(−/−)^ were similar to H_2_O_2-_treated WT parasites (Figure 2b, right panel). Mitochondrial superoxide levels measured by flow cytometry using MitoSOX were 2.7-fold higher in DDX3^(−/−)^ than in controls upon heat stress (Figure 2c), indicating an outburst of mitochondrial ROS in the absence of DDX3.

Increased ROS accumulation in DDX3^(−/−)^ may be attributed to a default in their detoxification. *Leishmania* encodes a mitochondrial iron superoxide dismutase (FeSODA) that detoxifies superoxide radicals into oxygen and hydrogen peroxide,^29^ which are further reduced by peroxidases.^30^ DDX3^(−/−)^ parasites showed a slight but significant increase in SOD activity that was higher in the presence of menadione, a known inducer of intracellular superoxide production (Figure 2d). On the other hand, peroxidase activity was decreased by 4-fold in DDX3^(−/−)^ cells upon induction with 0.6 mM H_2_O_2_ for 10 min compared with the controls (Figure 2e), indicating that *Leishmania* lacking DDX3 exhibit a defect in hydrogen peroxide detoxification.

### Inactivation of DDX3 causes mitochondrial membrane depolarization under stress leading to mitochondrial fragmentation and cell death

**Figure 3:**
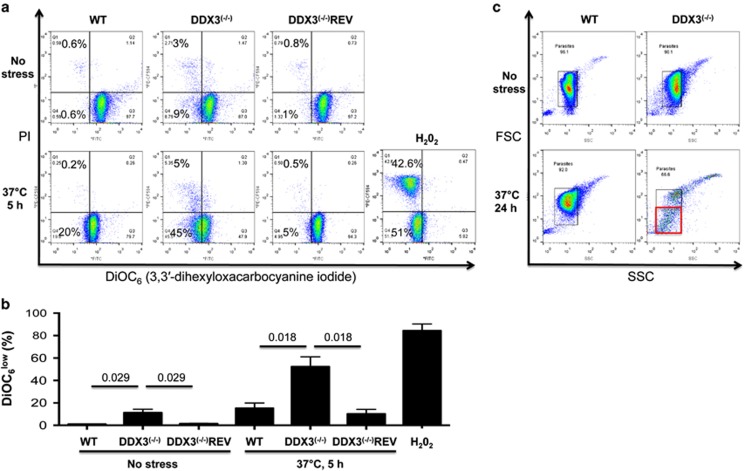
Heat stress induces mitochondrial membrane potential loss in *Leishmania* lacking DDX3. (**a**) 10^6^ parasites from WT, DDX3^(−/−)^, and DDX3^(−/−)^REV strains grown in RPMI medium were exposed or not to heat stress, double-stained with DiOC_6_(3) and propidium iodide (PI), and analyzed by flow cytometry to measure Δ*ψ*_m_ loss and membrane permeability and cell death, respectively. H_2_O_2_ treatment was used as a control inducing mitochondrial depolarization and death in ~50% of WT parasites after 8 h. (**b**) The results are means+S.E.M. of three independent experiments with duplicates and statistical significance was assessed using paired Student's *t*-test Prism version 6.0 (GraphPad Software, San Diego, CA, USA). The numbers over the lines correspond to *P-*values. (**c**) Cell morphology was assessed by flow cytometry on FSC and SSC parameters. WT and DDX3^(−/−)^ parasites were cultured either at room temperature or at 37 °C for 24 h. After O/N exposure to heat stress, most of the DDX3^(−/−)^ parasites demonstrated shrinkage (red square) and cell death

In *Leishmania* as well as in higher eukaryotes, increased ROS production results in inner mitochondrial membrane potential (Δ*ψ*_m_) loss, mitochondrial dysfunction, and cell death.^8, 31^ By flow cytometry, we measured Δ*ψ*_m_ loss using DiOC_6_ (3,3′-dihexyloxacarbocyanine iodide) and membrane permeability and cell death using propidium iodide (PI) under conditions of mitochondrial insults like prolonged heat-shock or oxidative stress. Even in the absence of stress, 9% of DDX3^(−/−)^ parasites exhibited a Δ*ψ*_m_ loss compared with 0.6 and 1% for WT and DDX3^(−/−)^REV, respectively (Figures 3a and b). Upon heat stress, the percentage of DDX3^(−/−)^ parasites with reduced Δ*ψ*_m_ increased up to 45% in comparison with 20% for WT and 5% for DDX3^(−/−)^REV (Figures 3a and b). Prolonged heat stress (24 h) elicited a significant decrease in the size of DDX3^(−/−)^ cells (Figure 3c). The shrinkage and granularity of DDX3^(−/−)^ parasites further infer that a population is subjected to necrosis or late apoptosis. As a positive control, WT parasites were treated (24 h) with H_2_O_2_, inducing massive mitochondrial membrane depolarization and cell death (Figures 3a and b).

**Figure 4:**
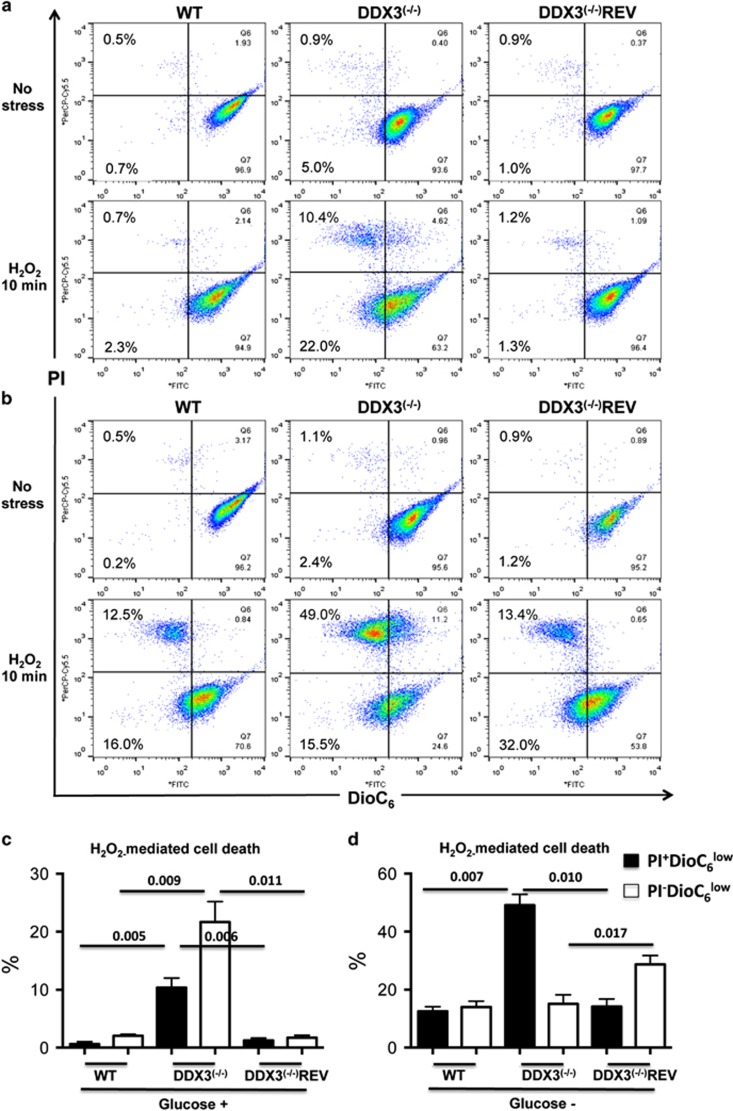
Mitochondrial membrane potential loss and cell death are greatly enhanced upon oxidative stress in DDX3^(−/−)^ *Leishmania* forced to use the mitochondrial oxidative respiratory machinery. (**a**) Flow cytometric analysis of DiOC_6_(3)- and PI-labeled *L. infantum* WT, DDX3^(−/−)^, and DDX3^(−/−)^REV grown in RPMI glucose-rich medium. Parasites were treated or not with 0.6 mM of hydrogen peroxide (H_2_O_2_) for 10 min, washed out, and further incubated in a fresh medium for 5 h prior to their analysis by flow cytometry. (**b**) As in (**a**) but parasites were cultured in DMEM glucose-free medium. The experiment was repeated three times with duplicates and statistical analysis of these results performed as in Figures 3b. (**c**) Glucose (+) medium) and (**d**) glucose (−) medium. The numbers over the lines correspond to *P-*values

A short exposure of DDX3^(−/−)^ parasites to H_2_O_2_ (0.6 mM for 10 min) induced Δ*ψ*_m_ loss (32.4% DiOC_6_^low^ parasites) and exacerbated cell death (10.4% DiOC_6_^low^PI^+^) (Figures 4a and c). To further investigate the impact of DDX3 on mitochondrial function, DDX3^(−/−)^ parasites were grown in glucose-free medium, hence forced to use the mitochondrial oxidative respiratory machinery (OXPHOS). Interestingly, Δ*ψ*_m_ loss was greatly increased in DDX3^(−/−)^ (64.5%) upon H_2_O_2_ stress resulting in a much higher percentage of dying cells (49% PI^+^) (Figures 4b and d) compared with parasites grown in glucose-rich medium (10.4% PI^+^) (Figures 4a and c). In DDX3^(−/−)^REV, despite a higher (2-fold) Δψ_m_ loss as compared with WT, the percentage of dying cells (PI^+^) remained similar to that of WT (13.4% *versus* 12.5%) (Figures 4b and d), suggesting that the add-back mutant is able to overcome mitochondrial stress in contrast to DDX3^(−/−)^ parasites (49% PI^+^ cells).

**Figure 5:**
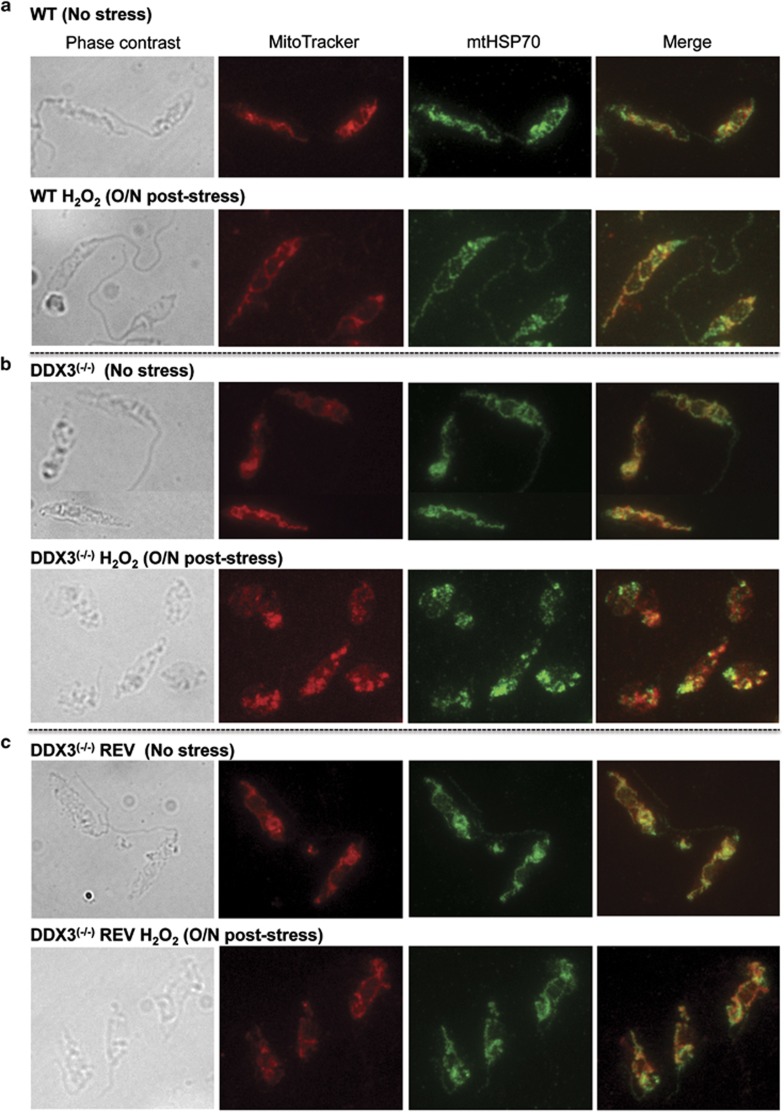
Inactivation of DDX3 impacts on mitochondrial morphogenesis upon exposure to oxidative stress. 2 × 10^7 ^*L. infantum* WT (**a**), DDX3^(−/−)^ (**b**), and DDX3^(−/−)^REV (**c**) grown under standard conditions (no stress) or treated for 10 min with 0.6 mM H_2_O_2_, washed out and further incubated O/N were stained with the MitoTracker CMX Red dye at a final concentration of 20 nM for 30 min at 26 °C. The mitochondrial network was visualized on 2% paraformaldehyde-fixed cells using a Nikon epifluorescence microscope. Immunolocalization studies were also performed using an anti-rabbit antibody against the mitochondrial matrix HSP70 protein. The data shown here are representative of three independent experiments that generated similar results

Because mitochondrial shaping and morphogenesis can be altered by apoptotic signals,^8^ we evaluated the mitochondrial network in DDX3^(−/−)^ and control parasites by fluorescence microscopy using the MitoTracker CMX Red dye. An antibody recognizing the mitochondrial matrix HSP70 protein was also used as a control. In the absence of DDX3, the mitochondrion appeared fragmented 24 h post-exposure to H_2_O_2_ (0.6 mM for 10 min) (Figure 5b, lower panel). In contrast, the WT (Figure 5a) and DDX3^(−/−)^REV (Figure 5c) strains recovered rapidly from oxidant stress, demonstrating a tubular mitochondrial shaping. These observations indicate that parasites lacking DDX3 display abnormal mitochondrial morphology upon oxidative stress in line with dissipation of the mitochondrial membrane potential and higher percentage of dead cells in stressed DDX3^(−/−)^ parasites (Figure 4).

Taken together, our results support a crucial role of DDX3 in mitochondrial morphogenesis and function, especially under conditions of mitochondrial stress.

### Depletion of DDX3 activates key components of the mitochondrial unfolded protein response

**Figure 6:**
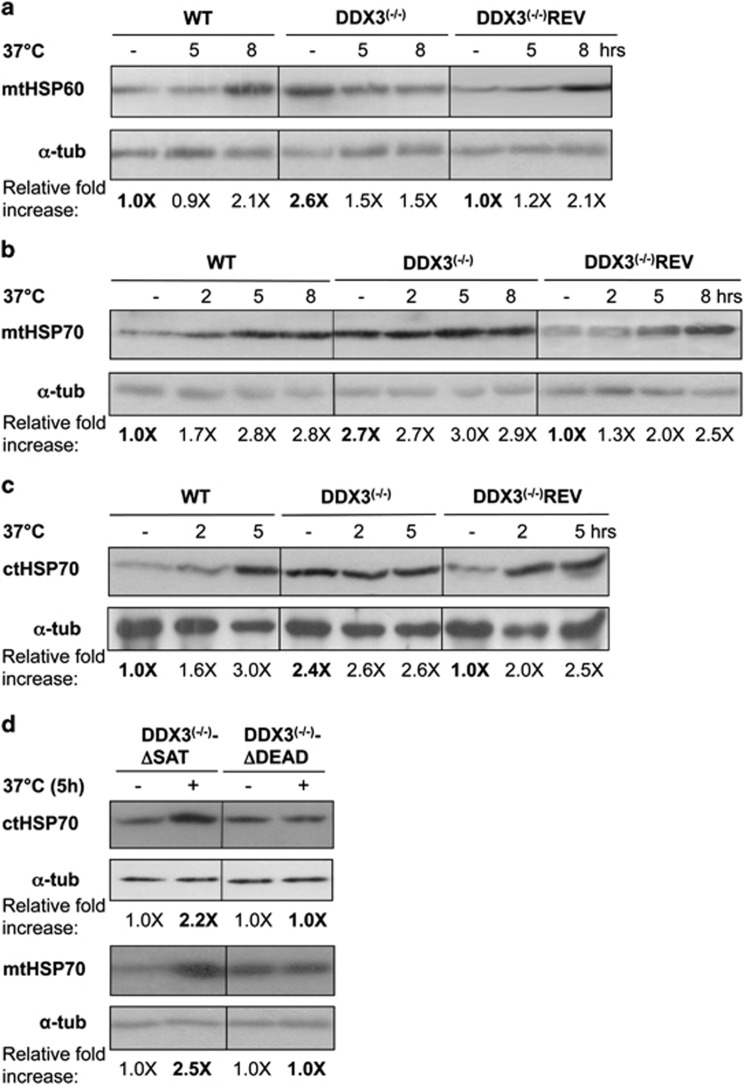
Inactivation of DDX3 activates key components of the mitochondrial unfolded protein response. Western blot analysis of total lysates from *L. infantum* WT, DDX3^(−/−)^ and DDX3^(−/−)^REV strains exposed to heat stress (37 °C) at different time points using an antibody recognizing the mitochondrial (mt) HSP60 (**a**) or mtHSP70 (**b**) or the cytoplasmic (ct) HSP70 (**c**) proteins (the same number of cells were loaded). Conditions of heat stress were compared with parasites grown at 25 °C. The same membrane was blotted with an anti-*α*-tubulin antibody (loading control). (**d**) Western blot analysis of total lysates from DDX3^(−/−)^ parasites complemented either with the DDX3ΔDEAD or the DDX3ΔSAT mutant proteins and subjected or not to heat stress (5 h) using an anti-ctHSP70 antibody. Data shown here are representative of three independent experiments yielding similar results. Relative fold increase was calculated from three experiments and within each strain (e.g., WT, DDX3^(−/−)^ and DDX3^(−/−)^REV) values correspond to the ratio of heat-stressed *versus* unstressed parasites normalized with the *α*-tubulin protein. Values in bold represent steady-state levels of mtHSPs in DDX3^(−/−)^ and DDX3^(−/−)^REV relative to WT levels (**a**–**c**). In panel (**d**), values in bold are relative to the unstressed control

Excessive ROS within mitochondria could disturb protein folding and trigger the mitochondrial unfolded protein response (UPR^mt^) to re-establish protein homeostasis.^18^ Mitochondria contain a dedicated repertoire of molecular chaperones in the matrix (e.g., mtHSP60 and mtHSP70) to facilitate protein folding and quality control proteases to degrade unfolded proteins.^18^ We examined baseline levels of mitochondrial HSP60 (Figure 6a) and HSP70 (Figure 6b) proteins by western blotting and found that they were 2.6- and 2.7-fold higher in DDX3^(−/−)^ cells, respectively compared with the WT and DDX3^(−/−)^REV. While WT parasites showed a time-dependent HSP60/HSP70 induction upon heat stress, no further increase was observed in DDX3^(−/−)^ cells under the same conditions (Figures 6a and b). A similar induction of mtHSPs was also observed in DDX3^(−/−)^ upon H_2_O_2_ stress (Supplementary Figure S2). Interestingly, the cytosolic HSP70, one of the most studied chaperones contributing to the cytosolic UPR,^32^ was also induced by 2.4/2.6-fold in DDX3^(−/−)^ in the absence of heat stress (Figure 6c). A mutant lacking the DEAD motif failed to restore HSP70 cytosolic and mitochondrial levels in DDX3^(−/−)^ whereas complementation with the DDX3ΔSAT mutant allowed a heat-stress-induced HSP70 response (Figure 6d) comparable to WT (Figure 6b). These results indicate that the UPR is activated in DDX3^(−/−)^ parasites, most likely in response to the increased load of mitochondrial ROS.

### DDX3 associates with major components of the cellular stress response and the mitochondrial protein quality control

To further dissect the role of DDX3 in mitochondrial function, we looked for potential DDX3-interacting proteins by immunoprecipitation (IP) studies followed by liquid chromatography-tandem mass spectrometry (LC-MS/MS). To assess reproducibility, IP experiments were repeated at least five times using two different methods, in gel digestion and on bead digestion, as indicated in Materials and Methods (Table 1 and Supplementary Table S1). In order to exclude nonspecific binders, we carried out two IP negative controls with total lysates from WT parasites (Supplementary Table S2) or recombinant *L. infantum* expressing GFP-HA (Supplementary Table S3) using an anti-HA antibody. A compilation from these experiments revealed potential interactions of DDX3 with key components of the cellular stress response. These include major antioxidant enzymes such as the iron superoxide dismutase (SODB1/SODB2) catalyzing the conversion of O_2_^•−^ to H_2_O_2_,^33^ tryparedoxin peroxidases involved in hydroperoxide detoxification^34^ and a member of the sestrin family (LinJ.18.0660) known to be central to ROS suppression in mammals.^35^ Also, several cytoplasmic and mitochondrial molecular chaperones and chaperonins (e.g., HSP40, HSP60, HSP70, HSP83, HSP110, and chaperonins containing T-complex protein 1) known to prevent nonspecific protein aggregates under stress^32^ were co-immunoprecipitated with DDX3.

Furthermore, DDX3 IP studies depicted p97 also called valosin-containing protein (VCP) in mammals and Cdc48 in yeast (p97/VCP/Cdc48; LinJ.36.1420) as a potential DDX3-interacting partner (Table 1). p97/VCP/Cdc48 is one of the best-characterized type II AAA+ (ATPases associated with various cellular activities) ATPases^36, 37^ shown to play an essential role in mitochondrial quality control by clearing oxidatively damaged mitochondrial membrane proteins through ubiquitination and proteasomal degradation.^21, 22, 23^ The interaction of DDX3 with p97/VCP/Cdc48 was confirmed by inverse co-IP studies using lysates from *L. infantum* expressing an HA epitope-tagged p97/VCP/Cdc48 (Table 1). Similarly to other eukaryotes,^38^ the *Leishmania* p97/VCP/Cdc48 homolog interacts with ubiquitin-activating and -degrading co-factors and adaptors (e.g., UFD1-NPL4, UBX- and UBA- or PUB-domain proteins) (Table 1 and Supplementary Table S4). In line with p97/VCP/Cdc48's role in the UPS, IP studies revealed potential interactions with proteasome subunits (RPT5, RPN1, RPN2, RPN5, RPN6, RPN8, RPN9), the AAA+ proteases FtsH and ClpB-type chaperone HSP78 known to selectively degrade damaged mitochondrial proteins,^18^ and prohibitins involved in stress tolerance and mitochondrial quality control^39^ (Table 1 and Supplementary Table S4). Thus, these data indicate that DDX3 associates with major components of the mitochondrial protein quality control in relationship with the UPS.

### DDX3 inactivation results in the accumulation of mitochondrial polyubiquitinated proteins

**Figure 7:**
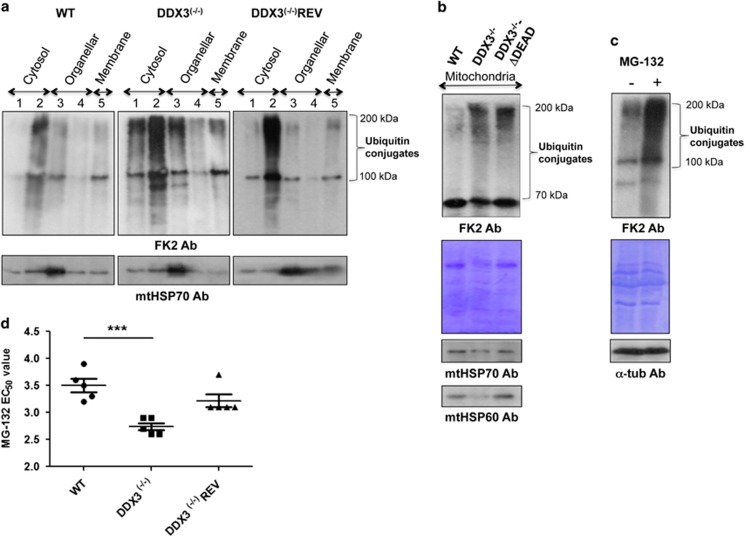
DDX3 inactivation results in the accumulation of mitochondrial polyubiquitinated proteins. (**a**) Digitonin (20 *μ*M–10 mM) fractions (equal volume) isolated from the same number of WT, DDX3^(−/−)^, and DDX3^(−/−)^REV parasites were analyzed by western blotting using the FK2 antibody recognizing K^29^-, K^48^-, and K^63^-linked mono- and polyubiquitinated proteins. As also determined by our previous experiments,^59^ digitonin fractions 1 and 2 are enriched with cytosolic proteins, fractions 3 and 4 with organellar material, and fraction 5 with membrane-associated proteins. An antibody directed against the mtHSP70 protein was used as a control to confirm mitochondrial enrichment in fraction 3. (**b**) Mitochondria purified from the same number of WT, DDX3^(−/−)^, and DDX3^(−/−)^-ΔDEAD (DDX3^(−/−)^ rescued with the DDX3ΔDEAD mutant) parasites using a 20–35% Percoll gradient centrifugation were analyzed by western blotting using the FK2 antibody. An equal amount of proteins was loaded on the gel. Shown here is one representative experiment out of three yielding similar results. (**c**) Western blot of total lysates from *L. infantum* WT treated with the proteasome inhibitor MG-132 (10 *μ*M) using the FK2 antibody. The anti-*α*-tubulin antibody and Coomassie blue staining were used as loading controls. (**d**) Susceptibility of WT, DDX3^(−/−)^, and DDX3^(−/−)^REV strains to MG-132.

The interaction of DDX3 with p97/VCP/Cdc48 prompted us to examine ubiquitinated protein levels in the mitochondrion of DDX3^(−/−)^ parasites as a measure of mitochondrial proteostasis. Mitochondrial proteins were enriched either by digitonin fractionation or by mitochondrial isolation using a 20–35% Percoll gradient. LC-MS/MS analysis confirmed a high enrichment of mitochondrial proteins in these fractions (data not shown). Western blotting using a monoclonal mono- and polyubiquitinated conjugates antibody (FK2) revealed higher levels of polyubiquitinated proteins in mitochondrial (lane 3) but also in cytosolic fractions (lanes 1 and 2) from DDX3^(−/−)^ compared with WT (lanes 1–3) and DDX3^(−/−)^REV (lanes 1 and 3) (Figure 7a). Increased levels of polyubiquitinated proteins were also observed in purified mitochondria from DDX3^(−/−)^ (Figure 7b). Similar results were obtained with DDX3^(−/−)^ parasites complemented with a DDX3 mutant lacking the DEAD motif (Figure 7b), indicating that altering the efficiency of DDX3 for binding to ATP is sufficient to induce accumulation of ubiquitinated proteins in the mitochondrion. As a control for protein ubiquitination, WT parasites were treated with 10 *μ*M of MG-132, a known proteasome inhibitor (Figure 7c). DDX3^(−/−)^ parasites were more sensitive to MG-132 compared with WT and DDX3^(−/−)^REV controls (Figure 7d).

## Discussion

DDX3 is a highly conserved member of the Ded1/DDX3 subfamily of DEAD-box RNA helicases that harbors ATPase and RNA helicase activities.^24^ Functionally, DDX3 appears to be one of the most multifaceted helicases with various roles in all steps of RNA metabolism.^24^ DDX3 is also implicated in cell cycle regulation, apoptosis, cell signaling, tumor progression or suppression, and viral infection.^40, 41^ Few reports demonstrated a role of DDX3 members in the response to stress and apoptosis. DDX3 was found in stress granules^42^ and shown to be involved in the response to hypoxia and radiation.^41, 43^ The *S. cerevisiae* ortholog Ded1 was found implicated in the response to nitrogen or glucose depletion, heat-shock, and low temperature.^44^ VBH-1 in *C. elegans* protects nematodes from heat-shock and oxidative stress.^45^

In this study, we describe a novel function for DDX3 in regulating the mitochondrial stress response and protein quality control. We show that *Leishmania* lacking DDX3 accumulate high levels of mitochondrial ROS and, upon stress, display mitochondrial membrane potential collapse associated with fragmentation. DDX3^(−/−)^ parasites are unable to recover from oxidative stress and experience cell death. Also, in the absence of DDX3, polyubiquitinated proteins accumulate in the cell, particularly in the mitochondrion, as an indicator of increased mitochondrial protein damage. In addition, we report potential interactions of DDX3 with key components of the mitochondrial stress response and particularly the AAA+ ATPase ubiquitin-selective chaperone p97/VCP/Cdc48, an essential component of mitochondrial protein quality control.

Our data support that DDX3 protects mitochondrial integrity from the damaged effects of ROS. Indeed, in the absence of DDX3, superoxide and hydrogen peroxide radicals accumulate in the mitochondrion upon the induction of various stresses due to a defect in their detoxification. Despite a slight increase in SOD activity, superoxide detoxification was largely impaired in DDX3^(−/−)^ parasites. Additionally, the dramatic decrease of peroxidase activity in this knockout strain further contributed to H_2_O_2_ accumulation and aggravated ROS-induced stress. In line with DDX3's role in the antioxidant stress response, immunoprecipitation studies revealed potential interactions of DDX3 with the mitochondrial iron superoxide dismutase SODB1/SODB2 and tryparedoxin peroxidases involved in the detoxification of superoxide and H_2_O_2_ radicals, respectively.^33, 34^ Mitochondrial tryparedoxin peroxidases have also been attributed a chaperone activity to ensure the integrity of mitochondrial functions.^46^ The association of DDX3 with proteins of the sestrin family sustains the idea that DDX3 can regulate ROS-induced mitochondrial stress. Sestrins are stress-inducible proteins known to suppress oxidative stress^35^ and their inactivation in invertebrates resulted in diverse metabolic pathologies and mitochondrial dysfunction.^47^

ROS accumulation can damage mitochondria inducing the collapse of mitochondrial membrane potential (Δ*ψ*_m_), mitochondrial dysfunction, and cell death.^48^ This is exactly what we have observed in DDX3^(−/−)^ *Leishmania* under conditions inducing mitochondrial stress and to a lesser degree in unstressed parasites. The mitochondrion was indeed severely damaged in DDX3^(−/−)^ parasites and appeared fragmented following oxidative stress. Shaping of mitochondria is essential for maintaining cellular bioenergetics by stabilizing the OXPHOS machinery and for regulating apoptosis.^8, 49^ The fact that DDX3^(−/−)^ parasites grown in glucose-free medium where cellular ATP is produced predominantly through OXPHOS exhibited a dramatic increase in mitochondrial depolarization and sensitivity to cell death upon oxidative stress, further supports a critical role of DDX3 in maintaining mitochondrial integrity. Increased ROS-mediated damage in DDX3^(−/−)^ parasites activated also the mitochondrial unfolded protein response^18^ through the induction of HSP70 and HSP60 levels in order to repair misfolded/damaged proteins. In line with these findings, our IP studies revealed DDX3 interactions with chaperones and chaperonins that are essential for mitochondrial function. HSPs were also found to facilitate protein degradation by the UPS, thus playing a role in the so-called ‘protein triage'.^50^

Consistent with these results is our finding that DDX3^(−/−)^ parasites accumulate polyubiquitinated proteins in the mitochondrion. Increased levels of polyubiquitinated proteins corroborate our IP studies revealing a potential interaction of DDX3 with the ubiquitin-selective chaperone p97/VCP/Cdc48. p97/VCP/Cdc48 is a cytosolic essential AAA+ chaperone that governs critical steps in ubiquitin-dependent protein quality control driving the turnover of misfolded polyubiquitinated proteins in different cellular compartments (e.g., ER, mitochondria, nucleus) by the 26 S proteasome.^22, 36, 37^ It has been shown recently that p97/VCP/Cdc48 is essential for the turnover of oxidatively damaged outer mitochondrial membrane (OMM) proteins.^21, 22, 23^ p97/VCP/Cdc48 is recruited to stressed mitochondria and upon ATP hydrolysis extracts ubiquitinated proteins from the OMM via its interaction with the UPS and presents them to the proteasome for degradation.^36, 51^ The interaction of DDX3 with p97/VCP/Cdc48 together with the increased accumulation of mitochondrial polyubiquitinated proteins in DDX3^(−/−)^ parasites support a role for DDX3 in mitochondrial protein quality control. Our finding that complementation of DDX3^(−/−)^ with a DDX3 mutant protein lacking the DEAD motif involved in the binding of the α and β phosphates of ATP^24, 52^ did not prevent accumulation of mitochondrial polyubiquitinated proteins suggests that the ATPase activity of DDX3 drives its function on mitochondrial proteostasis. It is possible that the interaction of DDX3 with p97/VCP/Cdc48 requires DDX3 binding to ATP and that this step is important for removing polyubiquitinated proteins out of the mitochondrion for proteosomal degradation. DDX3 does not seem to affect p97/VCP/Cdc48 binding to its co-factors or adaptors as IP studies against p97/VCP/Cdc48 in the DDX3^(−/−)^ background did not reveal any major changes in p97/VCP/Cdc48 interacting partners (Supplementary Table S5). Additional studies are needed, however, to fully address the impact of DDX3-p97/VCP/Cdc48 interaction on mitochondrial protein quality control.

Taken together, our data support a central role for the *Leishmania* DDX3 homolog in mitochondrial protein quality control under normal growth conditions and particularly upon stress by preventing ROS-mediated damage and polyubiquitinated protein accumulation in the mitochondrion. Because of its important role in tumorigenesis and viral infection,^40^ DDX3 represents a potent novel therapeutic target.^21^ On the other hand, defects in p97/VCP/Cdc48 activity have been linked to several human pathologies.^37, 53^ Accordingly, a comprehensive understanding of how DDX3 monitors mitochondrial quality control would possibly inform the prevention and treatment not only of parasitic but also of other human diseases.

## Materials and Methods

### Parasite strains and cell culture

*L. infantum* MHOM/MA/67/ITMAP-263 was used in this study. *L. infantum* promastigotes were cultured in SDM-79 medium supplemented with 10% heat-inactivated FCS (Multicell Wisent Inc., Canada) and 5 *μ*g/ml hemin at pH 7.0 and 25 °C. *L. infantum* axenic amastigotes were grown in MAA-20 medium supplemented with 20% FCS in 5% CO_2_ atmosphere at pH 5.5 and 37 °C.^54^ Bone marrow-derived murine macrophages were seeded in a 16-well chamber and infected with *L. infantum* as described previously.^55^

### Plasmid constructs and transfections

The *L. infantum DDX3* gene (LinJ.32.0410, http://tritrypdb.org) knockout and the rescue mutant DDX3^(−/−)^REV have been described previously.^6^ To engineer DDX3 deletion mutant proteins lacking either the DEAD-box (motif II) (DDX3ΔDEAD) or the SAT (motif III) (DDX3ΔSAT) or the HRIGRTGR motif (motif VI) (DDX3ΔHRIGRTGR), the Phusion DNA polymerase (NEB)-based PCR strategy was used. The HA-tagged DDX3 wild type and DDX3 mutant proteins were cloned into the *Xba*I and *Hin*dIII sites of vector pSPαZEOα.^56^ To generate the p97/VCP/Cdc48-HA construct, the *p97/VCP/Cdc48* ORF (LinJ.36.1420) was PCR-amplified from *L. infantum* and cloned into the *Hin*dIII and *Xba*I sites of pSP*α*ZEO*α*. Primer sequences or all constructs described here are indicated in Supplementary Table S6. Stable transfections of the above DNA constructs into *Leishmania* were carried out by electroporation as described previously.^57^

### Protein lysate preparations and western blots

Western blots were performed following standard procedures. The anti-mouse *α*-tubulin antibody (1:10 000 dilution; Sigma, Canada), the anti-mouse hemagglutinin (HA) tag monoclonal antibody (1:3000; ABM, Canada), the anti-HSP70 (cytosolic) monoclonal antibody universal (1:1000, (5A5)-Alexis Biochemicals, Canada), the anti-rabbit HSP70 (mitochondrial) antibody (1:2000, kindly provided by Dr Osvaldo de Melo Neto, Recife, Brazil), the anti-mouse HSP60 (mitochondrial) antibody (1:400; Acris, USA), and the anti-mouse mono- and polyubiquitinated conjugates monoclonal antibody (FK2) (1:5000 and blocking in 1% BSA; Enzo, Canada) were used in this study. FK2 Ab was used on total and digitonin-fractionated extracts as well as on purified mitochondria in the presence or absence of the proteasome inhibitor MG-132 (10 μM; Enzo). Mitochondria were purified from 5 to 10 × 10^9 ^log-phase *L. infantum* promastigotes using a 20–35% Percoll gradient, as described previously.^58^ Digitonin fractionation was carried out as described before.^59^

### ROS measurement

Intracellular oxidant species from *L. infantum* promastigotes were measured using 25 *μ*g/ml H_2_DCFDA flCFDA fluor probe (Invitrogen, Canada) and incubated at 25 °C for 30 min. Fluorescence was measured with a Victor fluorometer (Perkin-Elmer, USA), cells were counted, and RFU was normalized to 10^6^ cells. To monitor superoxide production, 10^6^ parasites grown in RPMI medium (Wisent, Canada) were stained for 20 min with the DHE (Life Technologies, Canada) probe and analyzed by flow cytometry. Mitochondrial superoxide was detected in live parasites using the fluorescent MitoSOX Red dye (Invitrogen). 2–4 × 10^7^ parasites were incubated with 5 *μ*M MitoSOX Red for 2 h at 25 °C and the fluorescence assessed by a Victor fluorometer (Perkin-Elmer, USA).

### Peroxidase activity

To measure peroxidase activity, we used the Peroxidase Activity Assay Kit (Sigma-Aldrich, Canada; MAK092) with some modifications. Briefly, stationary phase *L. infantum* promastigotes were treated with H_2_O_2_ (0.6 mM for 10 min), washed twice in PBS, and harvested by centrifugation at 6000 *g* for 10 min. The pellets were resuspended in 100 *μ*l of Assay Buffer, disrupted by several passages through a 30-gauge needle, and the lysate was centrifuged at 15 000 *g* for 10 min. Protein concentrations of parasite extracts were determined using the BCA protein assay system (Pierce, Canada). Peroxidase activity was measured via its coupling to H_2_O_2_ reduction and a fluorescent probe, resulting in a colorimetric (570 nm) product. Peroxidase activity reported as nmole/min/mg where one unit of peroxidase is defined as the amount of enzyme that reduces 1.0 mmol of H_2_O_2_ per minute at 37 °C.

### Superoxide dismutase activity

SOD activity was measured using OxiSelect Superoxide Dismutase Activity Assay (Cellbiolabs, USA; STA-340) according to the manufacturer's protocol. Stationary phase *L. infantum* promastigotes (10^9^) were treated or not with 3 μM menadione (Sigma) O/N, washed twice in PBS and harvested by centrifugation at 6000 *g* for 5 min. The pellets were resuspended in 100 *μ*l of cold 1 × Lysis Buffer (10 mM Tris, pH 7.5, 150 mM NaCl, 0.1 mM EDTA, protease inhibitor). Cells were lysed by three freeze–thaw cycles alternating liquid nitrogen and a 37 °C bath. The lysates were centrifuged at 15 000 *g* for 10 min at 4 °C, and protein contents were determined using a BCA protein assay system. The SOD assay system utilizes the xanthine oxidase, which oxidizes its substrate xanthine to produce superoxide anions. The included chromagen produces a water-soluble formazan dye upon reduction by superoxide anions. Inhibition of yellow formazan after the addition of whole-cell extracts to the SOD assay mix was determined as a measure of SOD activity. One unit of SOD will inhibit by 50% the rate of reduction of chromagen.

### Mitochondrial membrane potential measurement

The 3,3′-dihexyloxacarbocyanine iodide (DiOC_6_(3) (Molecular Probes, Eugenes, OR, USA) was used to measure mitochondrial membrane potential (Δ*Ψ*_m_) by flow cytometry. *L. infantum* promastigotes grown either in RPMI medium or in glucose-free medium (11966-DMEM; Life Technologies) were exposed or not to H_2_O_2_ treatment (0.6 mM for 10 min and further incubation in a fresh medium in the absence of oxidants), washed twice in PBS, and double-stained (10^6^ cells) with 40 nM DiOC_6_(3) for 20 min at RT and with l μg/ml of PI (Life Technologies) for 5 min on ice prior to flow cytometry analyses. DiOC_6_(3)/PI double-stained cells were analyzed on BD Influx (Becton Dickinson, Canada). Analyses were performed using FlowJo software (Tree Star, Inc., OR, USA), as well as GraphPad prism (GraphPad Software, Inc., San Diego, CA, USA) for statistics.

### MitoTracker staining and immunofluorescence

Parasites (2–4 × 10^7^/ml) were centrifuged and washed once with 1 × PBS and stained with MitoTracker CMX Red (Life Technologies) at a final concentration of 20 nM for 30 min at 26 °C. For immunofluorescence, cells were spotted on a slide (spread 5 μl), air dried at RT, washed twice in 1 × PBS and then fixed in 2% paraformaldehyde for 10 min. Following washings (3 ×) in 1 × PBS, cells were permeabilized in 1 × PBS, 0.2% Triton X-100, and 5% FCS at RT for 30 min and blocked in 1 × PBS, 2% milk, 0.02% Tween 20, and 0.1% Triton X-100 for 1 h at RT. The hybridization was performed with the anti-rabbit mitochondrial HSP70 (1:300, 1 h) followed by the secondary antibody, Alexa fluor 488 anti-rabbit (Life Technologies) (1:1000, 1 h). After washing, the cells were observed under a Nikon epifluorescence microscope.

### Immunoprecipitation studies and mass spectrometry analysis

For immunoprecipitation studies, *L. infantum* promastigotes expressing DDX3-HA or p97/VCP/Cdc48-HA proteins were lysed in ice-cold 1 × lysis buffer (10 mM Tris-HCl (pH 7.5), 50 mM KCl, 2 mM MgCl_2_, 1% Triton X-100, 10 *μ*l/ml of protease inhibitors (Sigma), protease inhibitor cocktail (Roche Applied Science, Canada), 1 mM DTT, 0.1 mM Na_3_VO_4_, 1 mM phenylmethylsulfonyl fluoride and 20 nM okadaic acid (Sigma)), passed through a 30-gauge needle and centrifuged at 10 000 *g* for 10 min at 4 °C. Soluble proteins (750 *μ*g) were mixed with Pierce Anti-HA Magnetic Beads (Thermo Scientific, Canada) as per the manufacturer's protocol. Proteins on beads were washed 3 × with 50 mM ammonium bicarbonate buffer and kept at −20 °C until mass spectrometry analysis. Alternatively, proteins eluted from beads were resolved only up to 1 cm in a 10% SDS PAGE followed by Coomassie blue staining. Then, 1 × 1 cm^2^ gel slice was excised, trypsin-digested, and analyzed by mass spectrometry. Peptide separation and elution, mass spectra acquisition, database searching, and peptide identification were performed as described previously.^60^ In order to exclude nonspecific binders to DDX3-HA or to p97/VCP/Cdc48-HA proteins, we carried out IP studies under the same experimental conditions with total lysates from either *L. infantum* wild type (Supplementary Table S2) or recombinant parasites expressing an HA-tagged GFP (Supplementary Table S3) as part of an episomal vector. Contaminant proteins present in the two IP negative controls were excluded from Table 1.

### Statistics

All analyses were performed using GraphPad Prism software (version 6.04; GraphPad, La Jolla, CA, USA). Results were expressed as means±S.D. or ±S.E.M. of at least three independent experiments in triplicates or in duplicates. Statistical significance was assessed by two-tailed paired Student's-*t*-test or the two-way ANOVA. Statistical significance was set at *P⩽*0.05 (**P⩽0.01, ***P⩽0.001, and ****P⩽0.0001).

## Figures and Tables

**Table 1:** Potentially interacting proteins with the *Leishmania infantum* DDX3 DEAD-box RNA helicase and the AAA-ATPase valosin-containing protein homolog p97/VCP/Cdc48 as determined by immunoprecipitation and LC-MS/MS studies

*TriTrypDB ID*	*Annotation*	*Peptide no.*^a^ *(probability score >95%)*	
		*WT*	
		*DDX3-HA*	*p97/VCP/Cdc48-HA*
**LinJ.32.0410**	**ATP-dependent RNA helicase HEL67 (DDX3)**	**21–41**	**5–22**

*Cell redox homeostasis/antioxidant activity*			
LinJ.32.1910/1920 LinJ.08.0300	Iron superoxide dismutase, putative (SODB1/SODB2) (mitochondrial) Iron superoxide dismutase, putative (FESODA) (mitochondrial)	3–7	2–4 2–3
LinJ.15.1140	Tryparedoxin peroxidase (TRYP)	2–10	6–7
LinJ.23.0050	Peroxidoxin, tryparedoxin peroxidase	2–6	1–6
LinJ.18.0660	PA26 p53-induced protein (sestrin), putative	2–2	
LinJ.05.0350	Trypanothione reductase (TRYR)		6–8
LinJ.27.1770	Trypanothione synthetase (TRYS)		7–10

*Protein folding–refolding*			
LinJ.28.2960	Heat-shock protein HSP70, putative	7–42	34–40
LinJ.30.2480	Heat-shock 70-related protein 1, mitochondrial precursor, putative	10–36	22–24
LinJ.26.1220	Heat-shock protein 70-related protein (HSP70.4)	2–9	18–23
LinJ.33.0360	Heat-shock proteins HSP83-2	3–38	30–31
LinJ.18.1350	Heat-shock protein HSP110, putative	1–30	14–20
LinJ.27.2350	Heat-shock protein DnaJ (HSP40), putative	2–8	9–10
LinJ.36.2130	Chaperonin HSP60, mitochondrial precursor	2–28	14–21
LinJ.23.1460	T-complex protein 1, gamma subunit (TCP-1-gamma), putative	1–13	4–14
LinJ.21.1330	T-complex protein 1, delta subunit (TCP-1-delta, putative	1–11	2–12
LinJ.35.3900	T-complex protein 1, eta subunit (TCP-1-eta), putative	3–9	4–11
LinJ.13.1400	Chaperonin TCP20, putative (TCP-1-zeta)	3–9	2–8

*Ubiquitin-dependent protein catabolic process/proteasome*			
**LinJ.36.1420**	**Valosin-containing protein, putative (p97/VCP/Cdc48)**	**4**–**8**	**37**–**59**
LinJ.35.1960	UBX domain containing protein, putative^b^		6–9
LinJ.22.0200	SEP domain containing protein, putative (NSFL1 p97 ATPase cofactor p47)^b^		7–13
LinJ.24.1650	Hypothetical protein, conserved (UBA-like, putative mitochondrial)^b^		3–8
LinJ.25.1320	NPL4 family, putative^b^		3–6
LinJ.09.1060	PUB domain containing protein, putative^b^		3–4
LinJ.11.0920	Short C-terminal domain/PUB domain containing protein, putative^b^		2–6
LinJ.36.6280	Hypothetical protein, conserved (ubiquitin thioesterase OTU1)^b^		1–4
LinJ.36.6780	Ubiquitin fusion degradation protein 1 (UFD1), putative^b^		7–12
LinJ.35.3110	Ubiquitin-activating enzyme E1, putative^b^		3–4
LinJ.09.0950	Polyubiquitin, putative		4–5
LinJ.12.0190	Proteasome regulatory ATPase subunittcc1l8.3 (AAA-ATPase)	3–6	
LinJ.22.0490	Proteasome regulatory ATPase subunit 5 (RPT5), putative (AAA-ATPase)		1–3
LinJ.29.0120	Proteasome regulatory non-ATPase subunit (RPN1), putative		5–6
LinJ.28.1850	Proteasome regulatory non-ATPase subunit 2 (RPN2), putative	2–2	3–6
LinJ.21.0840	Proteasome regulatory non-ATPase subunit 5 (RPN5), putative	2–3	2–7
LinJ.02.0340	Proteasome regulatory non-ATPase subunit 6 (RPN6), putative	3–4	2–6
LinJ.32.0400	19 S proteasome regulatory non-ATPase subunit 8 (RPN8), putative		1–2
LinJ.19.1100	Proteasome regulatory non-ATPase subunit 9 (RPN9), putative	1–4	2–5

*Mitochondrial integrity and stress response*			
LinJ.36.2850	ATP-dependent metallo-peptidase, Clan MA(E) (FtsH protease domain; AAA mitochondrial)^c^		12–19
LinJ.02.0680	ATP-dependent Clp protease subunit, HSP78, putative (AAA domain)^c^		3–3
LinJ.36.2850	ATP-dependent metallo-peptidase, Clan MA(E) (AAA/FtsH, mitochondrial)^c^		12–18
LinJ.35.1390	Mitochondrial metallo-peptidase, Clan ME		6–12
LinJ.05.1040	Stomatin-like protein, PHB prohibitin homolog^d^		14–20
LinJ.16.1710	Prohibitin (PHB1) (mitochondrial putative)^d^		5–8
LinJ.35.0070	Prohibitin (PHB2), putative (mitochondrial putative) (TM domains)^d^		7–12

a The proteins listed here co-immunoprecipitated with either the *Leishmania infantum* DDX3 homolog or the valosin-containing protein homolog p97/VCP/Cdc48 in 3–5 independent experiments with *L. infantum* DDX3-HA or p97/VCP/Cdc48-HA lysates. On bead or in gel digestion was followed by LC-MS/MS analysis (see the Materials and Methods). Proteins identified with a minimum of two peptides and a probability of >95.0% to correspond to the correct protein were included here. Identified proteins were grouped into different GO categories based on their predicted function. Only selected proteins related to the cellular stress response, the ubiquitin–proteasome system (UPS), and the mitochondrial protein quality control are indicated here. A detailed list of all co-immunoprecipitated proteins for more than three independent experiments with the protein, peptide, and spectrum reports is shown in Supplementary Tables S1 and S4.

b p97/VCP/Cdc48 co-factors/adaptors.^38^

c AAA+ mitochondrial proteases FtsH and ClpB-type chaperone HSP78 that selectively degrade misfolded and/or damaged mitochondrial proteins.^18^

d Prohibitins are inner mitochondrial membrane proteins that maintain mitochondrial functional integrity and protect cells from various stresses.^39^ Bold characters indicate the two major proteins used for the immunoprecipitation studies

## Abbreviations

ROS: reactive oxygen species
Δ_*Ψ*m_: mitochondrial membrane potential
UPS: ubiquitin–proteasome system
UPR: unfolded protein response
HSP: heat-shock protein
VCP: valosin-containing protein
AAA+: ATPases associated with diverse cellular activities
SOD: superoxide dismutase
DiOC_6_: 3,3′-dihexyloxacarbocyanine iodide
PI: propidium iodide
DHE: dihydroethidium
IP: immunoprecipitation
LC-MS/MS: liquid chromatography-tandem mass spectrometry
WT: wild type

## Supplementary Material

Supplementary Informations
