# Supplementary material for: DDX3 DEAD-box RNA helicase plays a central role in mitochondrial protein quality control in Leishmania
Source: Cell Death Dis. 2016 Oct 13;7(10):e2406–. doi: 10.1038/cddis.2016.315 (PMC5133982; doi:10.1038/cddis.2016.315)
Supplement: Supplementary Informations [file cddis2016315x1.docx]

**DDX3 DEAD-box RNA helicase plays a central role in mitochondrial protein quality control in *Leishmania***

**Prasad Kottayil Padmanabhan^1*^, Ouafa Zghidi-Abouzid^1*^, Mukesh Samant^2^, Carole Dumas^1^, Bruno Guedes Aguiar^1^, Jerome Estaquier^1,3^ and Barbara Papadopoulou^1#^**

**^1^**Research Center in Infectious Diseases, CHU de Quebec Research Center-University Laval and Department of Microbiology, Infectious Disease and Immunology, Faculty of Medicine, University Laval, Quebec, QC. Canada

^2^Department of Zoology, Kumaun University, Uttarakhand, India

^3^CNRS FR3636, Université Paris Descartes, Paris, France

*These authors contributed equally to this work.

# Corresponding author:

Barbara Papadopoulou

CHU de Quebec Research Center-University Laval

2705 Laurier Blvd., Quebec (QC), Canada G1V 4G2

Phone: (418) 525-4444, ext. 47608; Fax: (418) 654-2715

Email: [barbara.papadopoulou@crchul.ulaval.ca](mailto:barbara.papadopoulou@crchul.ulaval.ca)

Running title: DDX3 and mitochondrial proteostasis

**SUPPLEMENTARY MATERIAL**

**Supplementary figures**

**Figure S1. The *Leishmania* DEAD-box RNA helicase HEL67 shares all helicase core signature motifs with members of the eukaryotic DDX3 subfamily.**

(**a**) Sequence alignment of the *L. infantum* DEAD-box RNA helicase DDX3 homolog with the *Saccharomyces cerevisiae* Ded1 (NCBI, CAA99419), *C. elegans* VBH-1 (NCBI, NP_001021793), *Drosophila melanogaster* Belle (NCBI, AAF54262) and VASA (NCBI, NP_723899) and *Homo sapiens* DDX3 proteins (NCBI, NP_001347.3) by ClustalW multiple alignment using Bioedit sequence Alignment Editor. Gaps denoted by dashes have been introduced into the output by ClustalW in order to align the sequences. Identical amino acids in all sequences are shaded in black. The twelve major motifs of the DEAD-box protein helicase core are indicated with a red bar. The motif Q (selectivity for ATP), I (Walker A motif), II (LDEADRM or Walker B motif) and VI (HRIGRTGR) participate in ATP binding and hydrolysis. I and II motifs are specifically involved in the binding of the α and β phosphates of the ATP. The Ia, Ib (glycine duplets; not present in all SF2 members), Ic, III (SAT), IV, IVa, and V motifs are involved in RNA binding. The motif III has also been suggested to make the link between the ATPase and helicase activities. Similarly, the motif Va is important for the communication between ATP and RNA binding sites. The blue bars represent the residues deleted or mutated in the current study. (**b**) Unrooted phylogenetic tree of DEAD-box RNA helicases. Phylogenetic analysis was performed on the alignement of helicase core protein sequences from different species using Neighbor-joining statistical method. Branch lengths of the tree correspond to evolutionary distances computed using ones-Taylor-Thornton (JTT) model of the MEGA version 6 software. The topological accurancy of the tree was evaluated using 200 bootstrap replicates. The value on each branch is the estimated confidance limit (expressed as percentage) for the position of the branch as determined by bootstrap analysis. Only values exceeding 75% are shown. Members of the Ded1/DDX3 subfamily are indicated in blue and those of the Vasa/DDX4 subfamily in green. Protein names after the human DDX proteins denote the corresponding yeast ortholog. PTr–*Pan troglodytes*, HSa–*Homo sapiens*, MMu–*Mus musculus*, RNo–*Rattus norvegicus*, GGa–*Gallus gallus*, XTr–*Xenopus tropicalis*, DMe–*Drosophila melanogaster*, SCe–*Saccharomyces cerevisiae. Li*DDX3 (LinJ.32.0410) and *Li*Dbp1 (LinJ.35.3150) are the two *Leishmania* DDX3 homologs.

**Figure S2. H_2_O_2_ stress induces mitochondrial chaperone expression in DDX3^(-/-)^ *Leishmania*.**

Western blot analysis of total lysates from *L. infantum* WT, DDX3^(-/-)^ and DDX3^(-/-)^REV strains exposed or not to 0.6 mM H_2_O_2_ for 10 min. Measurements were taken at 5 and 8 hours post-stress using an antibody recognizing the mitochondrial (mt) HSP60 or mtHSP70 proteins (same number of cells were loaded). The same membrane was blotted with an anti-α-tubulin antibody (loading control). Data shown here are representative of two independent experiments yielding similar results. Relative fold increase was calculated from 2 experiments and within each strain (e.g., WT, DDX3^(-/-)^ and DDX3^(-/-)^REV) values correspond to the ratio of H_2_O_2_-stressed vs. unstressed parasites normalized with the α-tubulin protein. Values in bold represent steady-state levels of mtHSPs in DDX3^(-/-)^ and DDX3^(-/-)^REV relative to WT levels.

**Supplementary Table S1.** Protein, peptide and spectrum reports of LC-MS/MS analysis of *L. infantum* DDX3-HA immunoprecipitations.

**Supplementary Table S2.** Immunoprecipitation and LC-MS/MS peptide identification analysis of the control *L. infantum* wild type promastigotes using an anti-HA antibody.

**Supplementary Table S3.** Immunoprecipitation and LC-MS/MS peptide identification analysis of the control *L. infantum* GFP-HA expressing parasites using an anti-HA antibody.

**Supplementary Table S4.** Peptide and spectrum reports of LC-MS/MS analysis of *L. infantum* p97/VCP/Cdc48-HA immunoprecipitations.

**Supplementary Table S5.** Protein, peptide and spectrum reports of LC-MS/MS analysis of *L. infantum (Li)* p97/VCP/Cdc48-HA immunoprecipitations in the *Li*DDX3(-/-) background.

**Supplementary Table S6.** Primers used in this study.
