# Supplementary figures and images for: DDX3 DEAD-box RNA helicase plays a central role in mitochondrial protein quality control in Leishmania
Source: Cell Death Dis. 2016 Oct 13;7(10):e2406–. doi: 10.1038/cddis.2016.315 (PMC5133982; doi:10.1038/cddis.2016.315)

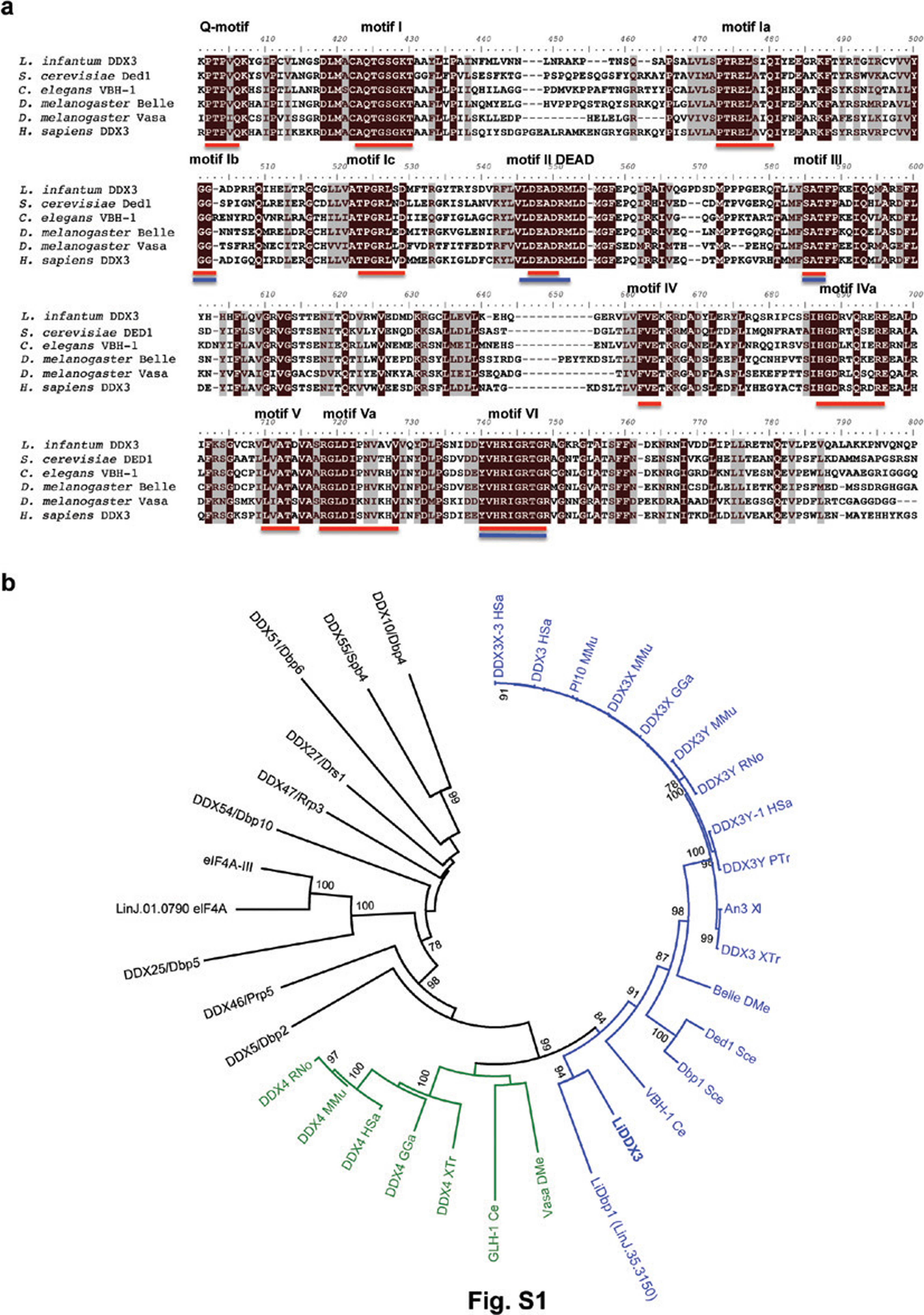

Supplement: Supplementary Figure S1 [file cddis2016315x2.tif]

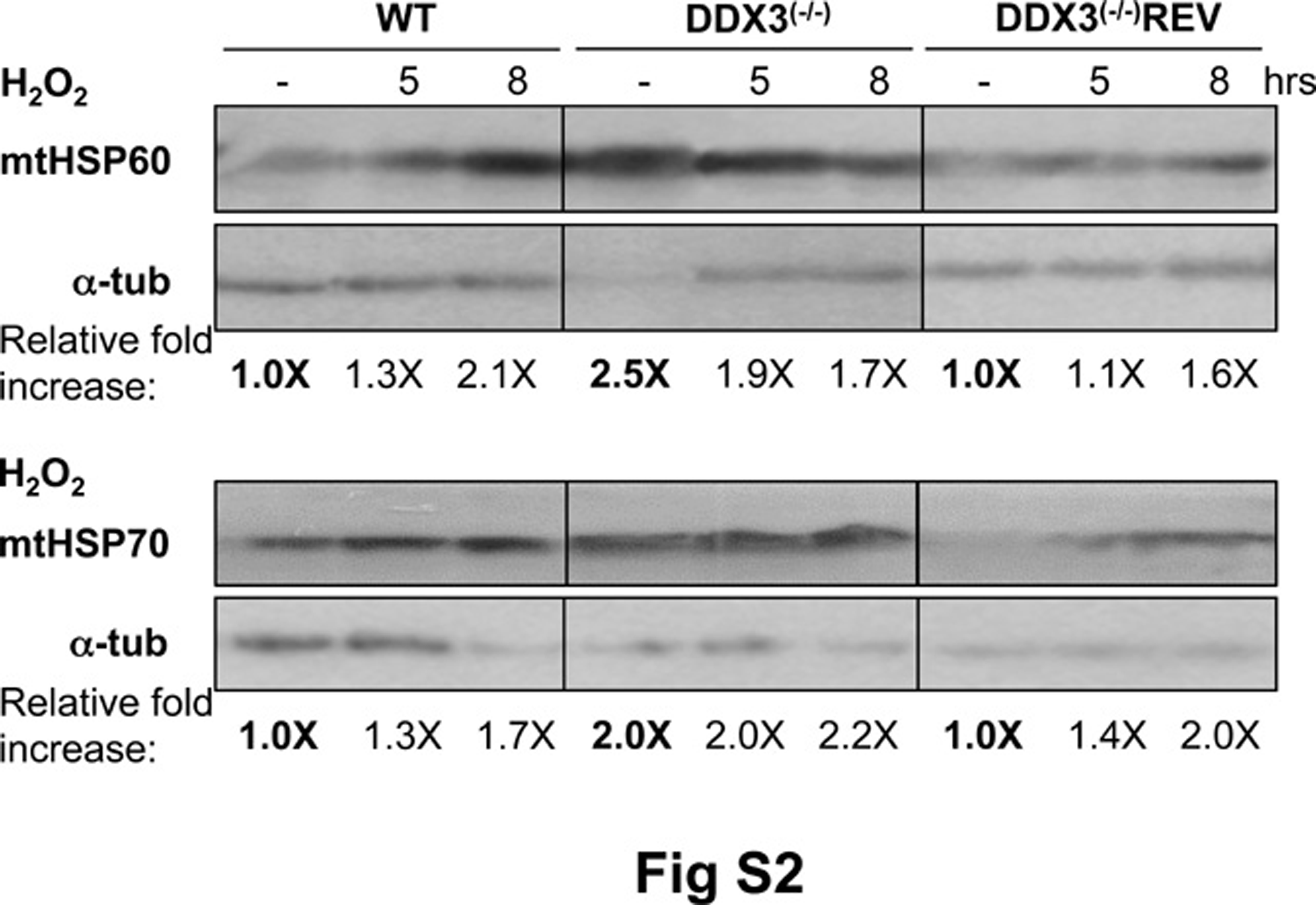

Supplement: Supplementary Figure S2 [file cddis2016315x3.tif]
