# Supplementary material for: DDX3 DEAD-box RNA helicase plays a central role in mitochondrial protein quality control in Leishmania
Source: Cell Death Dis. 2016 Oct 13;7(10):e2406–. doi: 10.1038/cddis.2016.315 (PMC5133982; doi:10.1038/cddis.2016.315)
Supplement: Supplementary Table S2 [file cddis2016315x5.docx]

**Supplementary Table S2.** Immunoprecipitation and LC-MS/MS peptide identification analysis of the control *L. infantum* wild type promastigotes using an anti-HA antibody.

| **TriTryp ID** | **Annotation** | **Molecular**  **Weight** | **No. of peptides** |
| --- | --- | --- | --- |
|  |  |  |  |
| LinJ.17.0090* | elongation factor 1-alpha | 49 kDa | 3 |
| LinJ.08.1280 | beta tubulin | 50 kDa | 2 |
| LinJ.13.0330 | alpha tubulin | 50 kDa | 2 |
| LinJ.13.1500* | programmed cell death 6 protein-like protein | 26 kDa | 2 |
| LinJ.18.0660 | hypothetical protein, conserved | 137 kDa | 3 |
| LinJ.36.6710 | protein transport protein sec23-like protein | 94 kDa | 2 |

*The four first proteins (highlighted in grey) were also identified with the *L. infantum* DDX3-HA immunoprecipitation and therefore were considered as contaminants and excluded from Table 1.
