# Supplementary material for: DDX3 DEAD-box RNA helicase plays a central role in mitochondrial protein quality control in Leishmania
Source: Cell Death Dis. 2016 Oct 13;7(10):e2406–. doi: 10.1038/cddis.2016.315 (PMC5133982; doi:10.1038/cddis.2016.315)
Supplement: Supplementary Table S3 [file cddis2016315x6.pdf]

Supplementary Table S3. Immunoprecipitation and LC-MS/MS peptide identification analysis of the control *L. infantum* GFP-HA expressing parasites[a] using an anti-HA antibody.

Accession Number		MW	GFP-HA[b]
**LinJ.32.0410**	**ATP-dependent RNA helicase, putative**	**67 kDa**	0
LinJ.07.0130	ATP-dependent DEAD/H RNA helicase, putative	53 kDa	0
LinJ.35.0370	ATP-dependent DEAD-box RNA helicase, putative	46 kDa	0
LinJ.29.2620	ATP-dependent phosphofructokinase	54 kDa	0
LinJ.35.3150	ATP-dependent RNA helicase, putative	101 kDa	0
LinJ.28.1420	ATP-dependent RNA helicase, putative	100 kDa	0
LinJ.35.4080	ATP-dependent RNA helicase, putative	79 kDa	0
LinJ.24.0240	ATP-dependent RNA helicase, putative	67 kDa	0
LinJ.05.0140	nucleolar RNA helicase II, putative	74 kDa	0
LinJ.28.2220	mitochondrial DEAD box protein, putative	60 kDa	0
LinJ.21.1820	RNA helicase, putative	59 kDa	0
LinJ.23.0240	ATP-binding cassette protein subfamily C, member 2, putative (ABCC2)	172 kDa	0
LinJ.23.0430	ATP-binding cassette protein subfamily G, member 5 (ABCG5)	135 kDa	0
LinJ.32.0790	RNA binding protein, putative (RPP25)	25 kDa	0
LinJ.35.2240	RNA-binding protein, putative	30 kDa	0
LinJ.25.0500	RNA-binding protein, putative, UPB1	27 kDa	0
LinJ.18.0590	RNA binding protein, putative	44 kDa	0
LinJ.30.0830	mitochondrial oligo_U binding protein TBRGG1, putative	75 kDa	0
LinJ.27.0980	mitochondrial RNA binding protein 1,gBP21, MRP1	27 kDa	0
LinJ.09.1180	mitochondrial RNA binding protein 2,MRP2, gBP25	27 kDa	1
LinJ.21.0600	la RNA binding protein, putative	37 kDa	0
LinJ.36.0050	PUF1, putative (PUF1)	61 kDa	0
LinJ.33.1210	pumilio protein 6, putative (PUF6)	92 kDa	0
LinJ.34.3010	RNA editing associated helicase 2, putative (REH2)	277 kDa	0
LinJ.36.2970	pre-mRNA splicing factor ATP-dependent RNA helicase, putative,dead/h helicase, putative	123 kDa	0
LinJ.07.1020	splicing factor ptsr1-like protein	42 kDa	0
LinJ.35.3030	chaperone protein DNAj, putative	51 kDa	0
LinJ.04.0940	chaperone protein DNAj, putative	52 kDa	0
LinJ.32.3500	chaperone protein DNAj, putative	45 kDa	0

LinJ.36.4690	chaperone protein DNAj, putative	31 kDa	0
LinJ.36.2130	chaperonin HSP60, mitochondrial precursor	61 kDa	0
LinJ.13.1400	chaperonin TCP20, putative	59 kDa	0
LinJ.36.0670	DNAJ protein-like protein	28 kDa	0
LinJ.26.0590	10 kDa heat shock protein, putative	11 kDa	0
LinJ.30.2480	heat shock 70-related protein 1, mitochondrial precursor, putative	72 kDa	0
LinJ.30.2470	heat shock 70-related protein 1, mitochondrial precursor, putative	69 kDa	0
LinJ.28.3040	heat shock protein 70, putative	72 kDa	0
LinJ.26.1220	heat shock protein 70-related protein (HSP70.4)	70 kDa	0
LinJ.33.0360	heat shock protein 83 (HSP83-2)	81 kDa	0
LinJ.33.0350	heat shock protein 83,heat shock protein 83-1 (HSP83-1)	79 kDa	0
LinJ.27.2350	heat shock protein DNAJ, putative	44 kDa	0
LinJ.28.2960	heat-shock protein hsp70, putative	71 kDa	4**
LinJ.23.1460	T-complex protein 1, gamma subunit, putative	60 kDa	0
LinJ.36.3360	14-3-3 protein-like protein	30 kDa	0
LinJ.14.1350	ubiquitin/ribosomal protein S27a, putative	17 kDa	0
LinJ.09.0950	polyubiquitin	98 kDa	0
LinJ.36.6600	ubiquitin-protein ligase, putative	454 kDa	0
LinJ.07.0440	ubiquitin-protein ligase-like, putative	671 kDa	0
LinJ.05.1140	vacuolar ATPase subunit-like protein	42 kDa	0
LinJ.36.0490	vacuolar protein sorting-associated protein, putative	69 kDa	0
LinJ.32.0970	vacuolar proton-ATPase-like protein, putative	101 kDa	0
LinJ.31.1240	vacuolar-type proton translocating pyrophosphatase 1, putative	83 kDa	0
LinJ.36.3250	ATP synthase, putative	25 kDa	0
LinJ.05.0500	ATPase alpha subunit	63 kDa	0
LinJ.25.1210	ATPase beta subunit, putative	56 kDa	0
LinJ.19.0190	ADP,ATP carrier protein 1, mitochondrial precursor, putative,ADP/ATP translocase 1, putative (ANC1)	35 kDa	0
LinJ.36.6180	kinetoplast DNA-associated protein, putative	18 kDa	0
LinJ.32.1200	kinetoplast polyadenylation/uridylation factor 2, putative	75 kDa	0
LinJ.30.3610	cytochrome p450-like protein	58 kDa	0
LinJ.32.2540	cytochrome-b5 reductase, putative	37 kDa	0
LinJ.18.1510	P-type H -ATPase, putative (H1A-2)	107kDa	0
LinJ.23.0050	peroxidoxin	25 kDa	0
LinJ.15.1100	tryparedoxin peroxidase (TRYP)	22 kDa	0
LinJ.28.2700	acyl-CoA dehydrogenase, putative	69 kDa	0

LinJ.21.0300	hexokinase	52 kDa	0
LinJ.36.1700	clathrin heavy chain, putative	191 kDa	79
LinJ.36.1040	40S ribosomal protein S10, putative	19 kDa	0
LinJ.13.0460	40S ribosomal protein S12, putative	16 kDa	0
LinJ.19.0390	40S ribosomal protein S13, putative	17 kDa	0
LinJ.28.1050	40S ribosomal protein S14	16 kDa	0
LinJ.22.0310	40S ribosomal protein S15, putative	17 kDa	1
LinJ.11.1180	40S ribosomal protein S15A, putative	15 kDa	0
LinJ.26.0840	40S ribosomal protein S16, putative	17 kDa	2
LinJ.28.2750	40S ribosomal protein S17, putative	16 kDa	1
LinJ.36.0990	40S ribosomal protein S18, putative	19 kDa	1
LinJ.34.2620	40S ribosomal protein S19 protein, putative	20 kDa	0
LinJ.29.2970	40S ribosomal protein S19-like protein	18 kDa	0
LinJ.19.0050	40S ribosomal protein S2	29 kDa	0
LinJ.32.0460	40S ribosomal protein S2	29 kDa	0
LinJ.11.0780	40S ribosomal protein S21, putative	32 kDa	0
LinJ.11.0770	40S ribosomal protein S21, putative	17 kDa	0
LinJ.21.1300	40S ribosomal protein S23, putative	16 kDa	0
LinJ.36.3010	40S ribosomal protein S24e (S24E-1)	16 kDa	0
LinJ.36.3940	40S ribosomal protein S27-1, putative	10 kDa	0
LinJ.15.1010	40S ribosomal protein S3, putative	24 kDa	0
LinJ.26.1610	40S ribosomal protein S33, putative (S33-1)	10 kDa	0
LinJ.35.0400	40S ribosomal protein S3A, putative	30 kDa	0
LinJ.13.1120	40S ribosomal protein S4, putative	31 kDa	0
LinJ.11.0960	40S ribosomal protein S5	21 kDa	0
LinJ.21.2150	40S ribosomal protein S6, putative (RPS6)	28 kDa	0
LinJ.24.2160	40S ribosomal protein S8, putative	25 kDa	0
LinJ.07.0760	40S ribosomal protein S9, putative	22 kDa	0
LinJ.36.5240	40S ribosomal protein SA, putative	28 kDa	0
LinJ.30.3780	60S acidic ribosomal protein P2, putative (LIP1)	10 kDa	0
LinJ.36.3950	60S ribosomal protein L10a, putative (RPL10a)	25 kDa	0
LinJ.29.2570	60S ribosomal protein L13, putative	25 kDa	0
LinJ.22.1370	60S ribosomal protein L14, putative	26 kDa	0
LinJ.13.0450	60S ribosomal protein L18, putative	22 kDa	0
LinJ.16.0470	60S ribosomal protein L21, putative	18 kDa	0

LinJ.36.3430	60S ribosomal protein L22, putative	15 kDa	0
LinJ.35.3840	60S ribosomal protein L23, putative	15 kDa	0
LinJ.06.0590	60S ribosomal protein L23a, putative	16 kDa	0
LinJ.24.2140	60S ribosomal protein L26, putative	16 kDa	0
LinJ.35.3830	60S ribosomal protein L27A/L29, putative	16 kDa	0
LinJ.11.1110	60S ribosomal protein L28, putative	16 kDa	0
LinJ.35.0240	60S ribosomal protein L30	11 kDa	0
LinJ.21.0800	60S Ribosomal protein L36, putative	12 kDa	0
LinJ.21.2190	60S ribosomal protein L37a, putative	10 kDa	0
LinJ.07.0550	60S ribosomal protein L7a, putative	39 kDa	0
LinJ.21.1290	60S ribosomal protein L9, putative	22 kDa	0
LinJ.29.1160	ribosomal protein L1a, putative	41 kDa	0
LinJ.36.1130	ribosomal protein L24, putative	15 kDa	0
LinJ.32.2830	ribosomal protein L27, putative	15 kDa	0
LinJ.32.3320	ribosomal protein L3, putative	48 kDa	0
LinJ.03.0240	ribosomal protein L38, putative	9 kDa	0
LinJ.20.1620	ribosomal protein S11 homolog	16 kDa	0
LinJ.28.1100	ribosomal protein S20, putative	13 kDa	0
LinJ.25.1220	ribosomal protein S25	13 kDa	0
LinJ.28.0570	ribosomal protein S26, putative	13 kDa	0
LinJ.28.2360	ribosomal protein S29, putative	7 kDa	0
LinJ.15.1530	ribosomal protein S6, putative,NPH2/RS6-like protein	14 kDa	0
LinJ.01.0430	ribosomal protein S7, putative	24 kDa	0
LinJ.20.1640	ribosome biogenesis protein, putative	36 kDa	0
LinJ.17.0090	elongation factor 1-alpha	49 kDa	14
LinJ.34.0870	elongation factor 1-beta (eEF1B beta 1)	26 kDa	0
LinJ.18.0740	elongation factor Tu, putative	52 kDa	0
LinJ.01.0790	eukaryotic initiation factor 4a, putative	45 kDa	0
LinJ.11.1160	eukaryotic release factor 3, putative	83 kDa	0
LinJ.27.1610	eukaryotic translation release factor, putative	51 kDa	0
LinJ.35.4200	poly(A)-binding protein 2,poly(a) binding protein, putative (PABP2)	65 kDa	0
LinJ.25.0080	poly(A)-binding protein 3, putative (PABP3)	61 kDa	0
LinJ.35.5360	polyadenylate-binding protein 1 (PABP1)	63 kDa	0
LinJ.32.1400	cleavage and polyadenylation specificity factor- like protein	167 kDa	0
LinJ.23.1030	hypothetical protein	102 kDa	0

LinJ.21.0490	hypothetical protein, conserved	47 kDa	0
LinJ.27.1220	hypothetical protein, conserved	60 kDa	0
LinJ.34.2410	hypothetical protein, conserved	22 kDa	0
LinJ.26.2000	hypothetical protein, conserved	39 kDa	0
LinJ.28.1890	hypothetical protein, conserved	30 kDa	0
LinJ.34.0560	hypothetical protein, conserved	42 kDa	0
LinJ.36.5330	hypothetical protein, conserved	103 kDa	0
LinJ.13.0270	hypothetical protein, conserved	13 kDa	0
LinJ.32.0020	hypothetical protein, conserved	56 kDa	0
LinJ.32.1150	hypothetical protein, conserved	179 kDa	0
LinJ.32.3040	hypothetical protein, conserved	33 kDa	0
LinJ.33.1020	hypothetical protein, conserved	66 kDa	0
LinJ.35.2330	hypothetical protein, conserved	54 kDa	0
LinJ.04.0830	hypothetical protein, conserved	146 kDa	0
LinJ.05.0450	hypothetical protein, conserved	22 kDa	0
LinJ.05.0580	hypothetical protein, conserved	86 kDa	0
LinJ.07.0140	hypothetical protein, conserved	91 kDa	0
LinJ.12.0014	hypothetical protein, conserved	101 kDa	0
LinJ.13.1030	hypothetical protein, conserved	65 kDa	0
LinJ.17.0010	hypothetical protein, conserved	88 kDa	0
LinJ.19.0020	hypothetical protein, conserved	28 kDa	0
LinJ.21.0620	hypothetical protein, conserved	11 kDa	0
LinJ.21.0880	hypothetical protein, conserved	249 kDa	0
LinJ.21.1420	hypothetical protein, conserved	29 kDa	0
LinJ.22.0180	hypothetical protein, conserved	61 kDa	0
LinJ.23.1790	hypothetical protein, conserved	28 kDa	0
LinJ.25.0720	hypothetical protein, conserved	39 kDa	0
LinJ.26.2040	hypothetical protein, conserved	39 kDa	0
LinJ.27.1630	hypothetical protein, conserved	38 kDa	0
LinJ.28.2070	hypothetical protein, conserved	72 kDa	0
LinJ.32.0890	hypothetical protein, conserved	56 kDa	0
LinJ.32.3630	hypothetical protein, conserved	86 kDa	0
LinJ.34.3420	hypothetical protein, conserved	18 kDa	0
LinJ.35.1080	hypothetical protein, conserved	64 kDa	0
LinJ.35.1510	hypothetical protein, conserved	65 kDa	0

LinJ.01.0420	hypothetical protein, conserved	68 kDa	0
LinJ.03.0830	hypothetical protein, conserved	250 kDa	0
LinJ.05.0610	hypothetical protein, conserved	103 kDa	0
LinJ.05.1180	hypothetical protein, conserved	212 kDa	0
LinJ.06.0570	hypothetical protein, conserved	183 kDa	0
LinJ.08.0390	hypothetical protein, conserved	202 kDa	0
LinJ.08.0820	hypothetical protein, conserved	55 kDa	0
LinJ.10.1140	hypothetical protein, conserved	33 kDa	0
LinJ.11.0150	hypothetical protein, conserved	94 kDa	0
LinJ.13.0440	hypothetical protein, conserved	71 kDa	0
LinJ.15.0890	hypothetical protein, conserved	89 kDa	0
LinJ.15.1280	hypothetical protein, conserved	46 kDa	0
LinJ.16.0090	hypothetical protein, conserved	83 kDa	0
LinJ.16.0440	hypothetical protein, conserved	20 kDa	0
LinJ.16.0520	hypothetical protein, conserved	126 kDa	0
LinJ.17.1120	hypothetical protein, conserved	72 kDa	0
LinJ.18.0820	hypothetical protein, conserved	290 kDa	0
LinJ.18.1150	hypothetical protein, conserved	107 kDa	0
LinJ.19.0430	hypothetical protein, conserved	132 kDa	0
LinJ.20.0770	hypothetical protein, conserved	171 kDa	0
LinJ.20.1130	hypothetical protein, conserved	128 kDa	0
LinJ.21.0440	hypothetical protein, conserved	25 kDa	0
LinJ.21.1460	hypothetical protein, conserved	146 kDa	0
LinJ.21.2220	hypothetical protein, conserved	109 kDa	0
LinJ.22.0006	hypothetical protein, conserved	66 kDa	0
LinJ.24.1420	hypothetical protein, conserved	44 kDa	0
LinJ.24.1810	hypothetical protein, conserved	43 kDa	0
LinJ.25.0380	hypothetical protein, conserved	20 kDa	0
LinJ.25.1920	hypothetical protein, conserved	72 kDa	0
LinJ.25.2520	hypothetical protein, conserved	109 kDa	0
LinJ.26.0700	hypothetical protein, conserved	213 kDa	0
LinJ.27.0220	hypothetical protein, conserved	35 kDa	0
LinJ.27.1870	hypothetical protein, conserved	177 kDa	0
LinJ.28.0640	hypothetical protein, conserved	10 kDa	0
LinJ.28.1440	hypothetical protein, conserved	38 kDa	0

LinJ.28.2120	hypothetical protein, conserved	81 kDa	0
LinJ.29.0250	hypothetical protein, conserved	59 kDa	0
LinJ.30.0140	hypothetical protein, conserved	38 kDa	0
LinJ.30.1600	hypothetical protein, conserved	93 kDa	0
LinJ.31.0240	hypothetical protein, conserved	100 kDa	0
LinJ.31.2450	hypothetical protein, conserved	55 kDa	0
LinJ.32.0150	hypothetical protein, conserved	111kDa	0
LinJ.32.0220	hypothetical protein, conserved	57 kDa	0
LinJ.32.1550	hypothetical protein, conserved	23 kDa	0
LinJ.32.2330	hypothetical protein, conserved	26 kDa	0
LinJ.32.2490	hypothetical protein, conserved	56 kDa	0
LinJ.32.3600	hypothetical protein, conserved	67 kDa	0
LinJ.32.3860	hypothetical protein, conserved	15 kDa	0
LinJ.32.4080	hypothetical protein, conserved	454 kDa	0
LinJ.33.0820	hypothetical protein, conserved	45 kDa	0
LinJ.33.1460	hypothetical protein, conserved	45 kDa	0
LinJ.33.1690	hypothetical protein, conserved	36 kDa	0
LinJ.34.3100	hypothetical protein, conserved	17 kDa	0
LinJ.35.0200	hypothetical protein, conserved	240 kDa	0
LinJ.35.1810	hypothetical protein, conserved	31 kDa	0
LinJ.35.4230	hypothetical protein, conserved	57 kDa	0
LinJ.35.4330	hypothetical protein, conserved	43 kDa	0
LinJ.36.0800	hypothetical protein, conserved	61 kDa	0
LinJ.36.3340	hypothetical protein, conserved	63 kDa	0
LinJ.36.3740	hypothetical protein, conserved	54 kDa	0
LinJ.36.5700	hypothetical protein, conserved	57 kDa	0
LinJ.36.5890	hypothetical protein, conserved	54 kDa	0
LinJ.36.6200	hypothetical protein, conserved	38 kDa	0
LinJ.36.6790	hypothetical protein, conserved	71 kDa	0
LinJ.36.7200	hypothetical protein, conserved	24 kDa	0
LinJ.23.1260	hypothetical protein, unknown function	73 kDa	0
LinJ.29.0300	hypothetical protein, unknown function	41 kDa	0
LinJ.12.0180	hypothetical protein, unknown function	113 kDa	0
LinJ.19.1020	hypothetical protein, unknown function	141 kDa	0
LinJ.30.1140	hypothetical protein, unknown function	263 kDa	0

LinJ.31.1050	hypothetical protein, unknown function	39 kDa	0
LinJ.31.2730	hypothetical protein, unknown function	60 kDa	0
LinJ.32.2660	hypothetical protein, unknown function	95 kDa	0
LinJ.35.1640	hypothetical protein, unknown function	39 kDa	0

Contaminant proteins obtained in the GFP-HA pull down.

[a] To generate a recombinant *L. infantum* GFP-HA cell line, the GFP-HA PCR-amplified fragment was cloned into HindII-XbaI sites of pGEM7Zf-αNEOα and this expression vector was transfected by electroporation into *L. infantum* wild type strain.

[b] GFP-HA pull down was compared with the *L. infantum* DDX3-HA immunoprecipitation experiments. Only 4 peptides were obtained for HSP70 vs. 7-42 in average in the *L. infantum* DDX3-HA IP experiment. All the other highly abundant heat-shock proteins and chaperonins (highlighted in grey) that were seen with the *L. infantum* DDX3-HA IP were not detected in the GFP-HA pull down. Similarly, the vast majority of ribosomal proteins found associated with the *L. infantum* DDX3-HA was not detected in the GFP-HA IP.
