# Supplementary material for: DDX3 DEAD-box RNA helicase plays a central role in mitochondrial protein quality control in Leishmania
Source: Cell Death Dis. 2016 Oct 13;7(10):e2406–. doi: 10.1038/cddis.2016.315 (PMC5133982; doi:10.1038/cddis.2016.315)
Supplement: Supplementary Table S6 [file cddis2016315x9.docx]

**Supplementary Table S6. Primers used in this study.**

| ***L. infantum* DDX3 mutant proteins** ^a^ |  | |
| --- | --- | --- |
| *Mutation of the motif II or DEAD box motif* | | |
| Primer A | 5’-CCCAAGCTTATGTATAAGAATCAGGCGCAAC-3’ | |
| Primer B | 5’-GCTCAAAGCCCATATCGAGTACGAGGAAGCGGACGTC-3’ | |
| Primer C | 5’-CTCGATATGGGCTTTGAGC-3’ | |
| Primer D* | 5’- CCCAAGCTTCTA**AGCGTAGTCTGGCACGTCGTAAGGGTA**CTGACCAAAGACGTCAGATCG-3’  HA-tag sequence | |
| *Mutation of the motif III (SAT)* | | |
| Primer A | 5’-CCCAAGCTTATGTATAAGAATCAGGCGCAAC-3’ | |
| Primer B | 5’-GCTGAATCTCCTTCGGGAAGTACAGCAGCGTCTGGCGCT-3’ | |
| Primer C | 5’-TTCCCGAAGGAGATTCAGC-3’ | |
| Primer D* | 5’- CCCAAGCTTCTA**AGCGTAGTCTGGCACGTCGTAAGGGTA**CTGACCAAAGACGTCAGATCG-3’  HA-tag sequence | |
| *Mutation of the motif VI* (*HRIGRTGR*) | | |
| Primer A | | 5’-CCCAAGCTTATGTATAAGAATCAGGCGCAAC-3’ |
| Primer B | | 5’-CGTACCGCGCTTGCCGGCCACATAGTCATCGATGTTGCTG-3’ |
| Primer C | | 5’-GCCGGCAAGCGCGGTACG-3’ |
| Primer D* | | 5’- CCCAAGCTTCTA**AGCGTAGTCTGGCACGTCGTAAGGGTA**CTGACCAAAGACGTCAGATCG-3’  HA-tag sequence |
| **p97/VCP/Cdc48 (LinJ.36.1420)-HA** | |  |
| LinJ.36.1420-P1 (Forward primer) | | 5’-GC**TCTAGA**ATGGCGGACGCTGTTGGG-3’ |
| LinJ.36.1420-P12 (Reverse primer) | | 5’-CCC**AAGCTT**TTA**AGCGTAGTCTGGCACGTCGTAAGGGTA**GCTGTAGAGGTCGTCGTCG-3’  HA-tag sequence |

### ^a^ To engineer DDX3 mutant proteins lacking amino acids corresponding to DEAD box (motif II), SAT (motif III) and HRIGRTGR (motif VI), the Phusion DNA polymerase-based PCR strategy was used. Primers A and B were used for amplifying the AB fragment and primers C and D for the amplification of the CD fragment from the *L. infantum DDX3* ORF. These fragments were fused in a single PCR using Phusion® High-Fidelity DNA Polymerase and primers A and D to generate fragment ABCD with deletions of selected amino acids. The PCR product with the desired mutation was purified from the gel and used in a second PCR reaction together with primer C harboring the HA tag epitope. The *DDX3* ORF was used as the template in both PCR reactions.
